# Replication and ribosomal stress induced by targeting pyrimidine synthesis and cellular checkpoints suppress p53-deficient tumors

**DOI:** 10.1038/s41419-020-2224-7

**Published:** 2020-02-07

**Authors:** Sona Hubackova, Eliska Davidova, Stepana Boukalova, Jaromira Kovarova, Martina Bajzikova, Ana Coelho, Mikkel G. Terp, Henrik J. Ditzel, Jakub Rohlena, Jiri Neuzil

**Affiliations:** 1 Laboratory of Molecular Therapy, Institute of Biotechnology, Czech Academy of Sciences, Prague-West, 252 50 Czech Republic; 2 Faculty of Science, Charles University, Prague, Czech Republic; 3 Department of Cancer and Inflammation Research, Institute of Molecular Medicine, University of Southern Denmark, 5000 Odense, Denmark; 4 Academy of Geriatric Cancer Research (AgeCare), Department of Oncology, Odense University Hospital, 5000 Odense, Denmark; 5 School of Medical Science, Griffith University, Southport, QLD 4222 Australia

**Keywords:** Cell biology, Cancer

## Abstract

p53-mutated tumors often exhibit increased resistance to standard chemotherapy and enhanced metastatic potential. Here we demonstrate that inhibition of dihydroorotate dehydrogenase (DHODH), a key enzyme of the de novo pyrimidine synthesis pathway, effectively decreases proliferation of cancer cells via induction of replication and ribosomal stress in a p53- and checkpoint kinase 1 (Chk1)-dependent manner. Mechanistically, a block in replication and ribosomal biogenesis result in p53 activation paralleled by accumulation of replication forks that activate the ataxia telangiectasia and Rad3-related kinase/Chk1 pathway, both of which lead to cell cycle arrest. Since in the absence of functional p53 the cell cycle arrest fully depends on Chk1, combined DHODH/Chk1 inhibition in p53-dysfunctional cancer cells induces aberrant cell cycle re-entry and erroneous mitosis, resulting in massive cell death. Combined DHODH/Chk1 inhibition effectively suppresses p53-mutated tumors and their metastasis, and therefore presents a promising therapeutic strategy for p53-mutated cancers.

## Introduction

Mitochondria are essential organelles with fundamental role in cellular metabolism and energy homeostasis. Besides being a major source of ATP, mitochondria also regulate cell death and differentiation, as well as cell cycle progression^1^. The mitochondrial oxidative phosphorylation system (OXPHOS) comprises respiratory complexes I–IV (CI–CIV) that are coupled to ATP generation catalyzed by complex V (CV). OXPHOS also involves dihydroorotate dehydrogenase (DHODH), a flavoprotein localized in the inner mitochondrial membrane. DHODH converts dihydroorotate (DHO) to orotate within the de novo pyrimidine synthesis pathway, generating electrons that are transferred, via redox-cycling of ubiquinone, to CIII^2^. Since DHODH is functionally linked to CIII activity, impairment of CIII function results in reduced activity of DHODH^3^. Hence, DHODH is an important link between de novo pyrimidine biosynthesis and mitochondrial respiration.

DHODH catalyzes conversion of DHO to orotate, a precursor of uridine monophosphate (UMP)^4^ that can yield all other pyrimidines. Since constant replenishment of the pyrimidine pool from glutamine via UMP is required for replication of nuclear DNA and cell growth, dysfunction of DHODH may result in genomic instability^5^, replication stress^6^, and cell cycle arrest^7^. This is particularly relevant for rapidly proliferating cancer cells that are not able to efficiently replenish their pyrimidine pool by salvage pathways and are therefore ‘addicted’ to DHODH-dependent synthesis of pyrimidines. Indeed, several DHODH inhibitors have been shown to suppress proliferation and to induce cell death in various types of tumors^6,8,9^.

While the tumor suppressor protein p53 plays a key role in DNA damage response, it is also a sensor of aberrant ribosomal biogenesis^10^. Its stability is controlled by the E3 ubiquitin ligase MDM2, which ubiqutinylates p53 to mark it for degradation. Under stress conditions, a number of ribosomal proteins, like RPL5 and RPL11, act as a p53 stabilizer^11,12^. Once stabilized, p53 modulates transcription of multiple target genes and ensures appropriate checkpoint maintenance, resulting in cell cycle arrest or apoptosis^13^. Since p53 serves as a tumor suppressor, the loss of wild-type (wt) p53 is beneficial for cancer cells. Notwithstanding the notion that more than 50% of human cancers lack functional p53, they are able to adapt to various types of stress^14^. Therefore, targeting p53-independent stress response pathways within p53-mutated background may be beneficial for cancer therapy.

Cell cycle checkpoints responding to DNA damage or incomplete DNA replication elicit cellular responses by inhibiting cyclin-dependent kinases that drive cell cycle progression, with checkpoint kinase 1 (Chk1) being a major checkpoint kinase in mammals^15^. Chk1 can be activated by ataxia telangiectasia and Rad3-related kinase (ATR) in response to DNA damage^16^ and regulates S phase entry and G2/M progression. As a consequence, cells are arrested at the checkpoints until damaged DNA is repaired^17^. Therefore, disruption of checkpoint signaling leads to re-entry of damaged cells into the cell cycle, resulting in cell death due to erroneous mitosis^18^. Besides its role in the maintenance of DNA integrity, Chk1 is a major regulator of cell cycle arrest after replication stress, independent of DNA damage^19^.

Here we demonstrate that Chk1 is activated in tumor cells as a consequence of replication/ribosomal stress after pyrimidine synthesis inhibition. Simultaneous inhibition of Chk1 and DHODH results in efficient elimination of p53-dysfunctional tumor cells in vitro as well as in suppression of tumor growth and metastatic dissemination in vivo. Such combination therefore represents a promising treatment strategy for patients with p53-dysfunctional cancer poorly responding to standard chemotherapy.

## Results

### Pyrimidine synthesis inhibition induces replication stress and cell cycle arrest

**Fig. 1:**
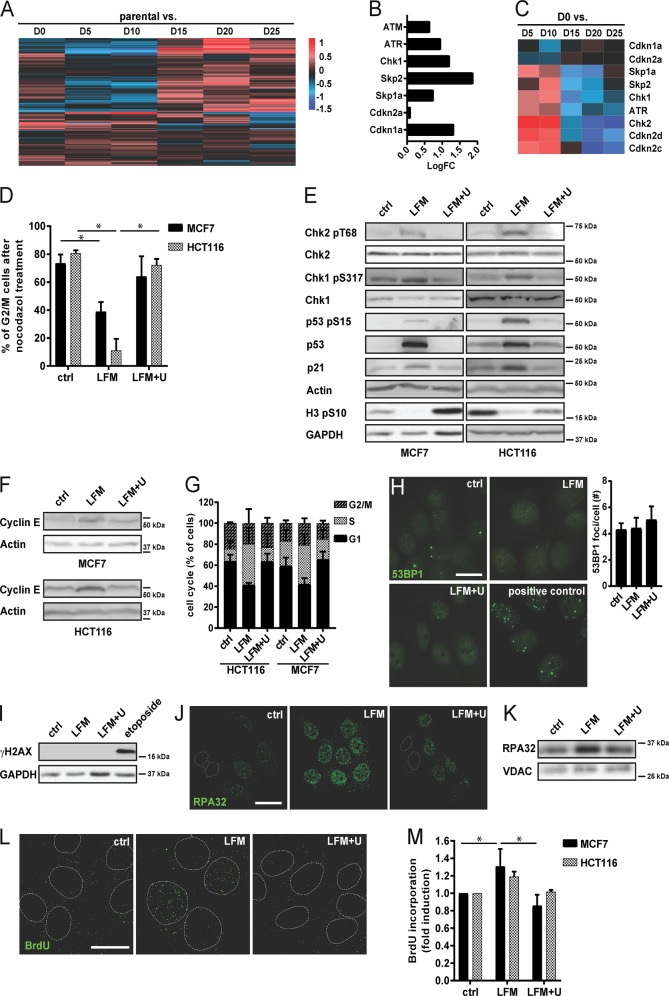
DHODH inhibition induces replication stress and cell cycle arrest. **a**–**c** Parental 4T1 cells and D0–D25 sublines were subjected to microchip analysis and heat map for the 194 transcripts involved in cell cycle and p53 signaling pathway was constructed. **d**–**m** MCF7 and HCT116 cells were exposed to leflunomide (LFM; 50 µM) for 72 h in the presence or absence of uridine (U; 50 µg/l). **d** Cells were incubated with nocodazole (10 µM, o/n) and the G2/M fraction was detected by flow cytometry using propidium iodide (2.5 µg/ml). **e** Immunoblotting detection of Chk2 pT68, Chk2, Chk1 pS317, Chk1, p53 pS15, p53, p21, and H3 pS10 and **f** cyclin E protein level was determined. β-Actin and GAPDH were used as a loading control. **g** MCF7 and HCT116 cells were assessed for cell cycle distribution. **h** Immunofluorescent detection of 53BP1 in MCF7 cells was performed. MCF7 cells treated with Etoposide (10 μM) for 24 h were used as a positive control. Scale bar, 10 µm. **i** Immunoblotting detection of γH2AX signal was performed in MCF7 cells. GAPDH was used as a loading control. **j** Accumulation of the RPA32 protein on DNA in MCF7 cells was evaluated by immunofluorescence staining and **k** by immunoblot analysis. VDAC was used as a loading control. **l** Accumulation of BrdU staining on non-denaturated ssDNA in MCF7 cells was evaluated by immunofluorescence staining and **m** by FACS. In **d**, **g** and **m**, data are shown as mean ± SEM, *n* = 3. *P < 0.05, two-way ANOVA. In other panels, representative experiment (from a total number of three experiments) is shown.

Recently we showed that reactivation of DHODH-linked pyrimidine biosynthesis is essential for tumor growth from mtDNA-depleted cancer cells. Restoration of DHODH activity, induced by respiration recovery following a horizontal transfer of mitochondria from the stroma, reinitiates pyrimidine synthesis, leading to the release of cell cycle arrest and to tumor proliferation^20^. To understand the molecular basis of cell cycle reactivation and its possible therapeutic potential, we performed transcriptome analysis of 4T1 sublines derived on various days after grafting of mtDNA-depleted 4T1ρ^0^ cells (referred to as day 0, D0 cells) in Balb/c mice from pre-tumor lesions (for detailed description see ref. ^20^). D5 and D10 cells were found to contain low levels of mtDNA derived from the host, reflected by compromised respiration. However, mitochondrial function as well as mtDNA levels was normalized in D15–D25 cells. Transcriptome analysis of cell cycle regulators revealed that D0–D10 cells, which are auxotrophic for uridine due to the lack of DHODH-dependent pyrimidine biosynthesis, clustered separately from D15 to D25 cells featuring fully restored DHODH-dependent de novo pyrimidine synthesis^20^ (Fig. 1a). Further analysis revealed an increased level of transcripts regulating cell cycle arrest (ATM, ATR, Chk1 or Cdkn1a) in D0–D10 time points as well as a decrease of transcripts linked to mitosis progression (Fig. 1b, Supplementary Tables 1 and 2). In addition, we observed a switch in this pattern when cells that are no longer auxotrophic for uridine (D15–D25 cells) were compared to D0–D10 cells (Fig. 1c, Supplementary Tables 1 and 2).

To understand the molecular mechanism of cell cycle arrest, we treated MCF7 and HCT116 cells with a DHODH inhibitor leflunomide for 72 h (Supplementary Fig. 1B, C), where we detected cell cycle arrest as well as increased β-galactosidase activity and a switch to senescence (Supplementary Fig. 1A). We observed decreased accumulation of cells in the G2/M phase in leflunomide-treated cells following synchronization with nocodazol (Fig. 1d), activation of the checkpoint kinases Chk1 and Chk2 by their phosphorylation, activation of the p53/p21 signaling pathway, decreased histone H3 phosphorylation on serine 10 (Fig. 1e), and increased β-galactosidase activity (Supplementary Fig. 1D). We observed similar results after silencing of DHODH using siRNA (Supplementary Fig. 1E–I). Overall, this points to a decrease in cell proliferation after DHODH inhibition. Supplementation of leflunomide-treated cells with uridine completely restored the parental phenotype, pointing to a link between suppression of pyrimidine synthesis and cell cycle arrest. Further, accumulation of cyclin E in leflunomide-treated cells indicates their arrest in early S phase (Fig. 1f), which is also supported by pulse BrdU incorporation in leflunomide-treated or DHODH-silenced cells (Fig. 1g, Supplementary Fig. 1F-H).

Checkpoint activation is critical for the induction of DNA damage response. However, we did not detect any DNA damage after DHODH inhibition, as assessed using the DNA damage markers 53BP1 and γH2AX (Fig. 1h, i, Supplementary Fig. 1J, K). The activation of checkpoints may therefore result from perturbation of the replication machinery, for example, as a consequence of pyrimidine depletion^21^. Using a pre-extraction protocol to remove unbound proteins, we observed increased association of single-strand DNA (ssDNA)-binding replication protein A (RPA^22^) with DNA in leflunomide-treated cells, which was reduced upon uridine supplementation (Fig. 1j, k, Supplementary Fig. 1L). Quantification of ssDNA in leflunomide-treated cells, detected by incorporation of BrdU under non-denaturated conditions, confirmed induction of replication stress following DHODH inhibition (Fig. 1l, m).

Interestingly, we recapitulated the same findings by silencing uridine monophosphate synthase (UMPS; Supplementary Fig. 2A), an enzyme catalysing formation of UMP from orotate, confirming the involvement of pyrimidine depletion and excluding non-specific effects of DHODH dysfunction in the OXPHOS system.

### Pyrimidine synthesis inhibition stabilizes the p53 tumor suppressor protein

**Fig. 2:**
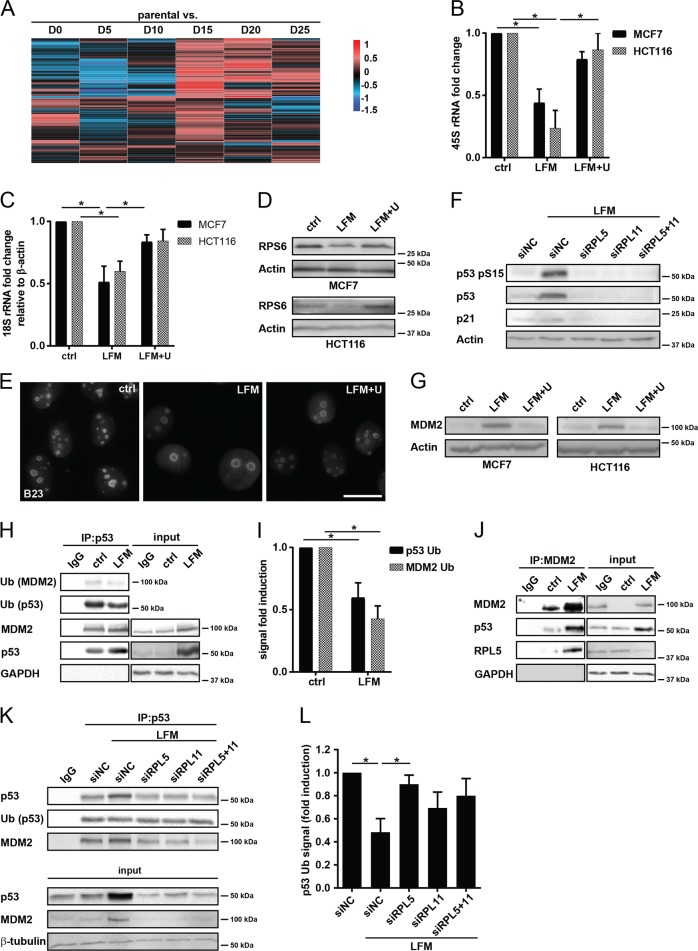
Inhibition of DHODH stabilizes the p53 tumor suppressor protein. **a** Parental 4T1 cells and D0–D25 sublines were subjected to microchip analysis and a heat map for the 119 transcripts involved in ribosome biogenesis was constructed. **b**–**e**, **g** MCF7 and HCT116 cells were exposed to leflunomide (LFM, 50 µM) for 72 h in the presence or absence of uridine (U; 50 µg/l). Expression of 45S rRNA (**b**) and 18S rRNA (**c**) was assessed by qRT-PCR. **d** Immunoblotting detection of RPS6 was performed. β-Actin was used as a loading control. **e** Immunofluorescent detection of B23 in MCF7 cells was performed. **f** Immunoblotting detection of p53 pS15, p53, and p21 in MCF7 cells treated 72 h with LFM (50 µM) after downregulation of RPL5 and RPL11 alone or in combination using specific siRNA. Non-targeting siRNA (siNC) was used as a control. β-Actin was used as a loading control. **g** Immunoblot detection of MDM2 in MCF7 and HCT116 cells. β-Actin was used as a loading control. **h** Co-immunoprecipitation of p53 followed by immunoblot analysis of p53, MDM2, ubiquitinated p53, and ubiquitinated MDM2 in MCF7 control and LFM (50 µM, 72 h)-treated cells. GAPDH was used as an input control. Immunoprecipitated samples and input represent one membrane with different intensity of signal. **i** Levels of ubiquitinated p53 and MDM2 related to their immunoprecipitated total forms were evaluated from three independent experiments. **j** Co-immunoprecipitation of MDM2 followed by immunoblot analysis of MDM2, p53, and RPL5 protein in MCF7 control and LFM (50 µM, 72 h)-treated cells. GAPDH was used as an input control. Immunoprecipitated samples and input represent one membrane with different intensity of signal. **k** Co-immunoprecipitation of p53 followed by immunoblot analysis of total and ubiquitinated p53 and MDM2 in MCF7 cells treated with LFM (50 µM, 72 h) after downregulation of RPL5 and RPL11 alone or in combination using specific siRNA. Non-targeting siRNA (siNC) was used as a control. p53, MDM2, and β-tubulin were used as an input control. **l** The level of ubiquitinated p53 related to its immunoprecipitated total form was evaluated from three independent experiments. In **b**, **c**, **i**, and **l**, data are shown as mean ± SEM, *n* = 3. *P < 0.05, two-way ANOVA. In other panels, representative experiment (from total number of three experiments) is shown.

Persistent DNA replication stress^23^ and, importantly, depletion of the pyrimidine nucleotide pool, especially UTP, resulting from DHODH inhibition, could impair biogenesis of ribosomes, which activates the p53 pathway leading to cell cycle arrest^10^. Similarly as for the cell cycle, we detected decreased levels of ribosomal transcripts in uridine-dependent D0–D10 cells and their normal levels in proliferating D15–D25 cells (Fig. 2a, Supplementary Table 3). We examined ribosomal synthesis by measuring 45S rRNA, a precursor rRNA which is processed into mature 28S, 18S, and 5.8S rRNAs that form ribosomal subunits. A decreased level of 45S rRNA and 18S rRNA was observed in MCF7 and HCT116 cells after their treatment with leflunomide (Fig. 2b, c) or after UMPS silencing (Supplementary Fig. 2B). Consistent with this, we found reduced mRNA and protein level for RPS6, a crucial ribosomal component responsible for docking of mRNAs onto the ribosome (Fig. 2d, Supplementary Fig. 2C; see also Supplementary Table 3 for the RPS6 transcript). To demonstrate the importance of functional ribosomes for cell survival, we silenced RPS6 in MCF7 cells, which resulted in increased cell death (Supplementary Fig. 2D, E) and p53 stabilization (Supplementary Fig. 2F). Moreover, increased nucleolar fusion connected with ribosomal stress (Fig. 2e for MCF7, Supplementary Fig. 2G for HCT116) confirmed defects in ribosomal biogenesis in leflunomide-treated cells^24^. Similarly, as for the cell cycle arrest, uridine supplementation completely reverted the phenotype. Together, these results point to the induction of ribosomal stress after inhibition of DHODH.

Perturbation of ribosome biogenesis can sometimes lead to disruption of nucleoli^25^, followed by translocation of ribosomal proteins such as RPL5 and RPL11 from the nucleolus to the nucleoplasm, where they bind to MDM2 and inhibit its ubiquitin ligase activity required for p53 degradation^26^. To investigate the role of RPL5 and RPL11 in p53 stabilization after DHODH inhibition, we silenced both proteins either alone or in combination. This resulted in reduced leflunomide-induced p53 stabilization and decreased induction of its downstream target p21 in the silenced cells (Fig. 2f for MCF7, Supplementary Fig. 2H for HCT116; for siRNA efficacy see Supplementary Fig. 2I).

MDM2 is normally degraded by the proteasome together with the p53 protein. To demonstrate this process, blocking of MDM2 binding to p53 by Nutlin3, an MDM2 inhibitor, resulted in stabilization of both proteins and cell cycle arrest in our experimental system (Supplementary Fig. 2J–L). Since MDM2 was increased in leflunomide-treated cells (Fig. 2g), we tested its ability to bind p53. Using immunoprecipitation, we detected decreased ubiquitination of p53 and MDM2 after leflunomide treatment rather than changes in the MDM2-p53 interaction (Fig. 2h, i). Moreover, we confirmed binding of the RPL5 protein to MDM2 after inhibition of DHODH (Fig. 2j). Silencing of RPL5 and RPL11 then restored ubiquitination of p53 (Fig. 2k, l) and its subsequent degradation (Fig. 2f, Supplementary Fig. 2H), documenting that these RPL proteins released by ribosomal stress induced by DHODH inhibition interfere with MDM2-mediated ubiquitination of p53 and promote its stabilization.

### p53 deficiency sensitizes to cell death upon leflunomide treatment

**Fig. 3:**
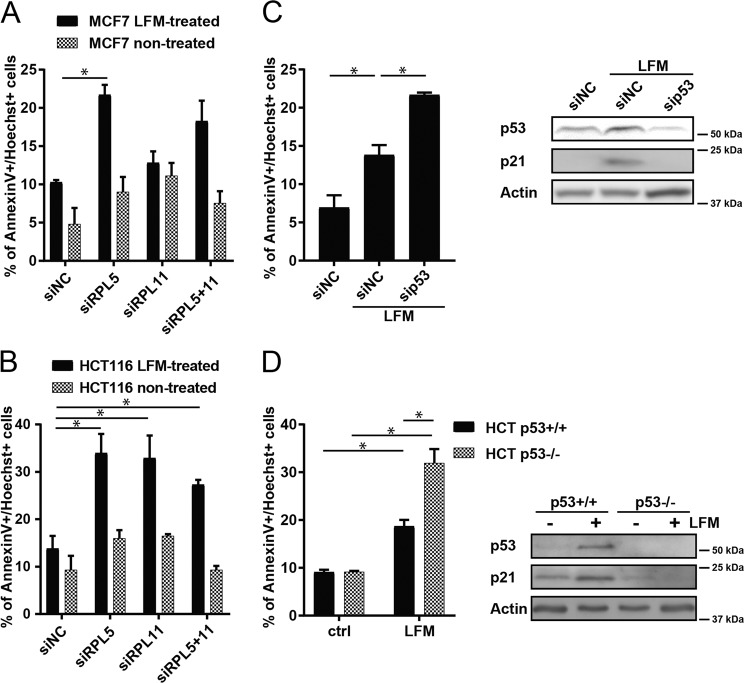
Role of p53 in cell cycle arrest after leflunomide treatment. **a** MCF7 and **b** HCT116 cells were exposed to LFM (50 µM) for 72 h after downregulation of RPL5 and RPL11 alone or in combination using specific siRNA. Cell death was assessed by flow cytometry using annexin V/Hoechst staining. Non-targeting siRNA (siNC) was used as a control. **c** MCF7 cells were transfected with specific p53 siRNA or with non-targeting siRNA (siNC), respectively. Cell death was evaluated by annexin V/Hoechst positivity using FACS. Immunoblot detection of p53 and p21 shows the effect of transfection. β-Actin was used as a loading control. **d** HCT116 wt p53 and HCT116 p53^KO^ cells were exposed to LFM (50 µM) for 72 h and cell death was determined by flow cytometry using annexin V/Hoechst staining. Immunoblot detection of p53 and p21 show the effect of p53^KO^. β-Actin was used as a loading control. In **a**–**d**, data are shown as mean ± SEM, *n* = 3-5. *P < 0.05, two-way ANOVA.

Aberrant execution of the cell cycle in the absence of effective checkpoints can lead to cell death. Searching for the role of RPL in cell death, we observed that RPL silencing increased cell death upon leflunomide treatment (Fig. 3a, b, Supplementary Fig. 3C–E; see Supplementary Fig. 3A, B for siRNA efficacy). To provide direct evidence that p53, stabilized by RPL proteins, promotes cellular viability in leflunomide-treated cells, we knocked down p53 in MCF7 cells and used these (Fig. 3c, Supplementary Fig. 3F, G) as well as HCT116 p53^KO^ cells (Fig. 3d, Supplementary Fig. 3H) to show that in both cases, p53 deficiency promots cell death upon leflunomide treatment. We conclude that the loss of functional p53 synergizes with DHODH inhibition in cell death induction, indicating an important role of p53 in regulating cellular decision between cell cycle arrest and cell death after impairment of de novo pyrimidine synthesis.

### Pyrimidine synthesis inhibition induces cell cycle arrest independently of p53

**Fig. 4:**
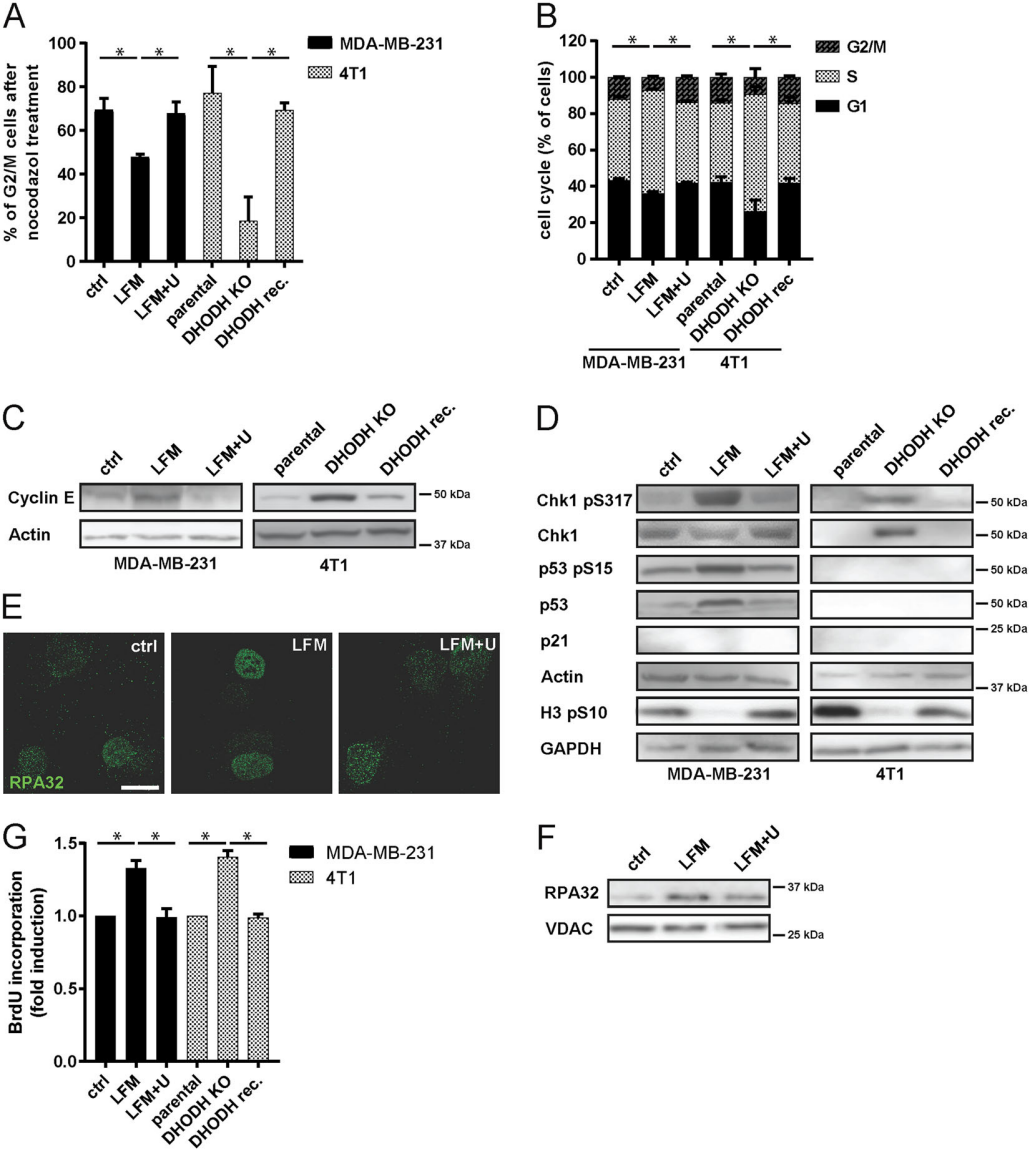
DHODH inhibition induces cell cycle arrest in p53-deficient cells. **a**–**g** MDA-MB-231 cells were exposed to LFM (50 µM) for 72 h in the presence or absence of uridine (U; 50 µg/l). **a** Control and treated MDA-MB-231 cells as well as 4T1 parental, DHODH knock-out (KO) and DHODH reconstituted (rec.) cells were treated with nocodazole (10 µM, o/n) and analyzed for G2/M arrest using propidium iodide (2.5 µg/ml) staining detected by flow cytometry. **b** MDA-MB-231 and 4T1 cells were assessed for cell cycle distribution. The asterisk indicates significantly different values for S-phase changes. **c** Cyclin E and **d** Chk1 pS317, Chk1, p53 pS15, p53, p21, and H3 pS10 protein levels were detected by immunoblot. β-Actin and GAPDH were used as a loading control. **e** Accumulation of RPA32 protein in MDA-MB-231 cells was evaluated by immunofluorescence and **f** immunoblot. VDAC was used as a loading control. Scale bar represents 15 μm. **g** Accumulation of BrdU staining on non-denaturated ss-DNA in MDA-MB-231 cells was evaluated by FACS. In **a**, **b**, and **g**, data are shown as mean ± SEM, *n* = 3. *P < 0.05, two-way ANOVA. In other panels, representative experiment (from total number of three experiments) is shown.

Using p53-mutant MDA-MB-231 breast cancer cells treated with leflunomide and p53-deficient 4T1 breast cancer cells with ablated DHODH (DHODH^KO^ cells; see Supplementary Fig. 4A, B for DHODH activity), we observed a similar effect of pyrimidine depletion on the cell cycle as found for wt p53 cells (Fig. 4a; see Fig. 1a for wt p53 cells) with arrest in early S-phase (Fig. 4b, c, Supplementary Fig. 4C), increased β-galactosidase activity (Supplementary Fig. 4D), and checkpoint activation (Fig. 4d). Similarly, we detected accumulation of the RPA protein (Fig. 4e, f) and BrdU (Fig. 4g) on ssDNA as a result of impaired replication, as well as perturbation of ribosomal biogenesis and ribosomal stress (Supplementary Fig. 4G-I) independent of DNA damage, which was not detected (Supplementary Fig. 4E, F). Uridine supplementation or re-expression of DHODH in DHODH^KO^ 4T1 cells restored the parental phenotype. These results reveal that while p53 stabilization induces cell cycle arrest, the block in cell cycle progression upon DHODH inhibition can be p53-independent, pointing to an additional mechanism of cell cycle inhibition.

### Inhibition of Chk1 promotes cell death in DHODH-inhibited p53-deficient tumor cells

**Fig. 5:**
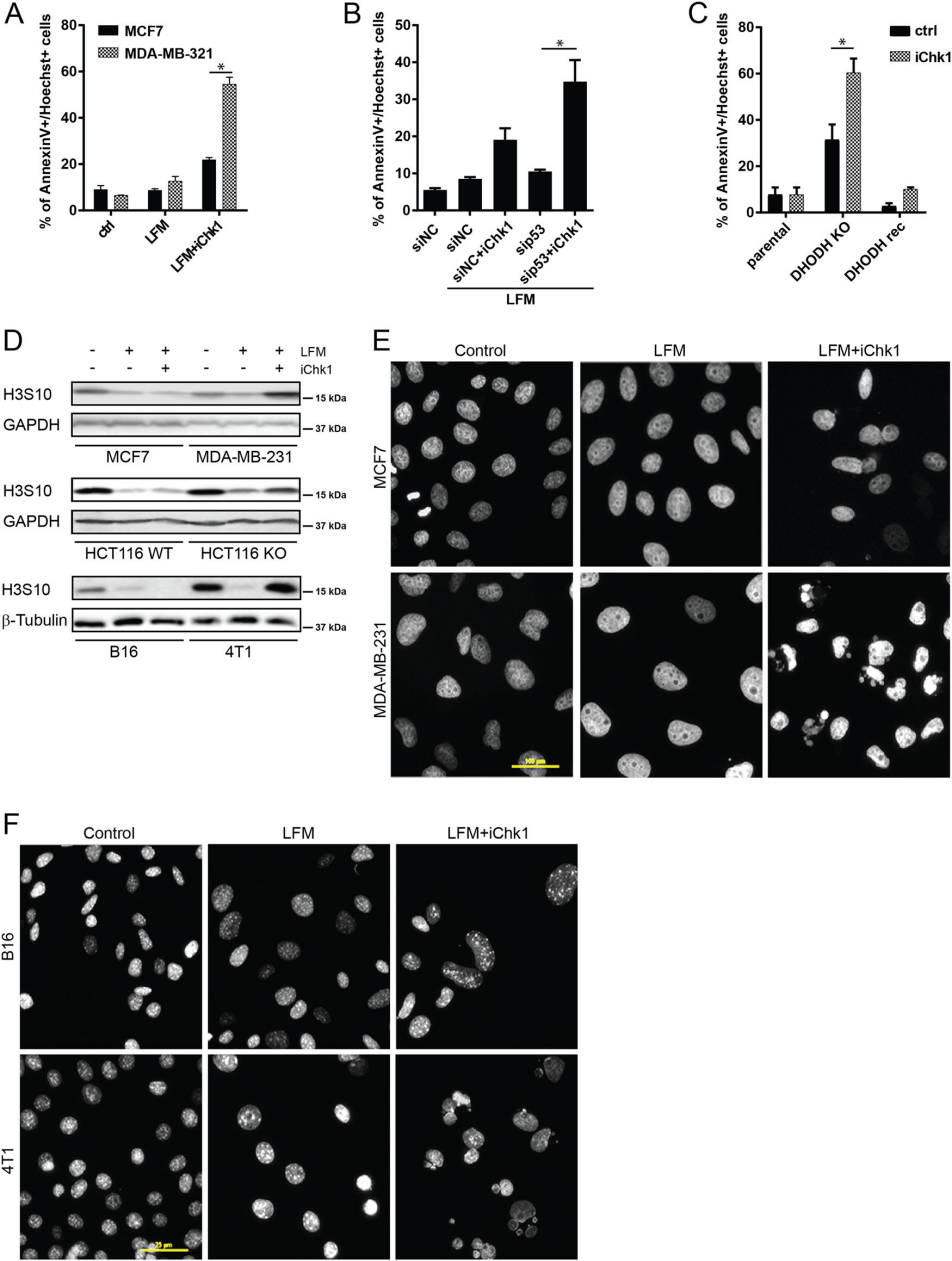
Inhibition of Chk1 promotes cell death in DHODH-inhibited p53-deficient tumor cells. **a** MCF7 and MDA-MB-321 cells were pre-treated with Chk1 kinase inhibitor LY2603618 (iChk1, 5 µM, 30 min before LFM) followed by LFM treatment (50 µM, 48 h) and cell death was evaluated by annexin V/Hoechst staining using flow cytometry. **b** MCF7 cells were pre-treated with iChk1 (5 µM, 30 min before LFM) after downregulation of p53 using specific siRNA followed by LFM treatment (50 µM, 48 h). Cell death was detected by annexin V/Hoechst staining using FACS. **c** 4T1 parental, DHODH knock-out (KO), and DHODH reconstituted (rec) cells were treated with iChk1 (5 µM, 48 h) and cell death was evaluated by annexin V/Hoechst staining using FACS. **d**–**f** MCF7, MDA-MB-321, HCT116 wt p53, HCT116 p53^KO^, B16, and 4T1 cells were pre-treated with iChk1 (5 µM, 30 min before LFM) followed by LFM treatment (50 µM, 24 h). **d** Protein level of H3 pS10 was detected by immunoblot. GAPDH or β-tubulin was used as a loading control. **e**, **f** Nuclear morphology was detected by a fluorescent microscope using DAPI staining. Scale bar represents 100 µm (**e**) or 25 µm (**f**). In **a**–**c**, data are shown as mean ± SEM, *n* = 3. *P < 0.05, two-way ANOVA. In other panels, representative experiment (from total number of three experiments) is shown.

Chk1 activation after DHODH inhibition (Figs. 1e and 4d) as well as the ability of p53-deficient or p53-mutant cells to induce cell cycle arrest indicates its potential role in the response to pyrimidine depletion. Using MCF7 and MDA-MB-231 cells, we observed increased cell death upon Chk1 inhibition by LY2603618 in leflunomide-treated cells (Fig. 5a, Supplementary Fig. 5B; for Chk1 inhibition efficacy see Supplementary Fig. 5A). Surprisingly, this effect was much stronger in p53-mutant MDA-MB-231 cells compared to p53-wt MCF7 cells. To investigate whether p53 deficiency sensitizes cells to Chk1 inhibition, we silenced p53 in MCF7 cells (Supplementary Fig. 5C), which resulted in elevated cell death upon Chk1 inhibition in response to leflunomide treatment (Fig. 5b, Supplementary Fig. 5D). To verify these findings in an independent model, we examined HCT116 p53^KO^ cells (Supplementary Fig. 5E, F). Furthermore, we compared mouse p53-deficient 4T1 and NeuTL cells with wt p53 B16 mouse cells (Supplementary Fig. 5G, H). Using these additional models, we also observed a strong correlation between p53 deficiency and sensitization to combined Chk1/DHODH inhibition. Finally, to control for possible off-target effects of leflunomide and to demonstrate directly that DHODH deficiency sensitizes to Chk1 inhibition, DHODH^KO^ 4T1 cells were tested. Similarly to leflunomide-treated cells, we detected increased cell death in DHODH^KO^ cells exposed to the Chk1 inhibitor (Fig. 5c, Supplementary Fig. 5I), which was reverted when DHODH activity was restored by DHODH reconstitution (Supplementary Fig. 4A).

Activation of checkpoints prevents damaged or stressed cells to progress through the cell cycle. Inhibition of these checkpoints could therefore induce a mitotic catastrophe arising as a result of aberrant re-entry of tumor cells into the cell cycle^27^. While phosphorylation of histone H3 at serine 10 (H3 pS10), an epigenetic mitotic marker^28^, was found downregulated in tumor cells treated with leflunomide independent of the p53 status (Fig. 5d), its increased levels were observed in p53-deficient cells (4T1 cells and p53^KO^ HCT116 cells) or p53-mutant MDA-MB-231 cells after Chk1 inhibition (Fig. 5d), indicating re-entry of these cells into the cell cycle. Also, while nuclei of wt p53 cells (MCF7 and B16 cells) remained unchanged, we observed robust nuclear fragmentation in p53-mutant or p53-deficient cells (MDA-MB-231 and 4T1 cells, respectively) after combined treatment with leflunomide and the Chk1 inhibitor (Fig. 5e, f) as a consequence of cell cycle progression without replicated DNA.

We therefore demonstrate here that depressed pyrimidine synthesis caused by inhibition of DHODH activates, via p53 and Chk1, two alternative checkpoint pathways that contribute to cell cycle arrest. This explains sensitivity of p53-deficient cells to Chk1 inhibition resulting in aberrant mitosis and cell death.

### Simultaneous Chk1 and DHODH inhibition sensitizes p53-deficient tumors to cell death and blocks metastases

**Fig. 6:**
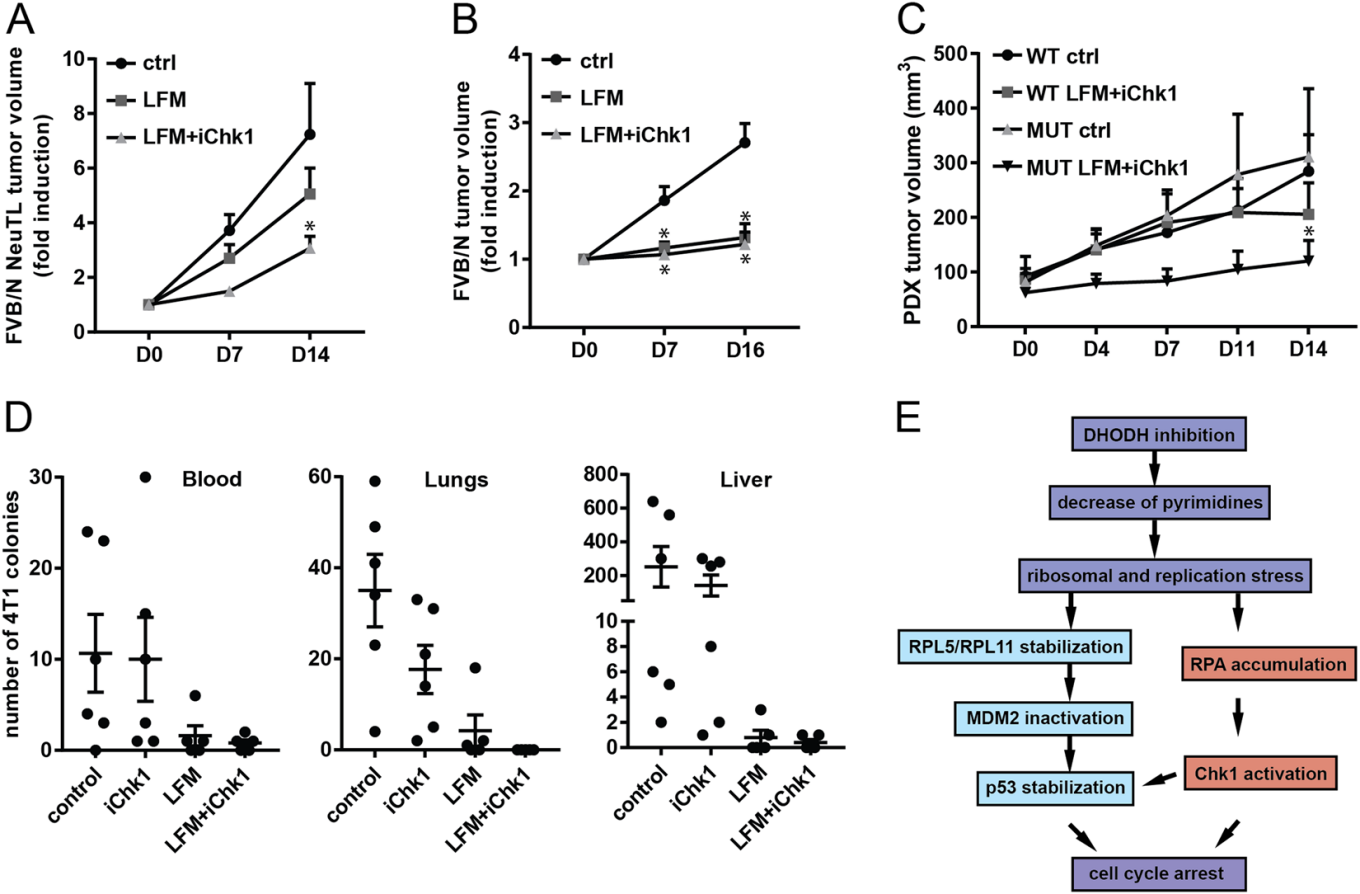
Simultaneous Chk1 and DHODH inhibition sensitizes p53-deficient tumors to cell death and blocks metastases. **a** FVB/N *c-neu* mice subcutaneously injected with syngeneic NeuTL cells (1 × 10^6^ cells per animal; 5 mice per group) and **b** FVB/N *c-neu* mice (three mice per group) with spontaneous tumors were treated intraperitoneally with LFM (20 mg/kg) alone or in combination with iChk1 (20 mg/kg)—see Methods for details. Tumor volumes were evaluated. **c** NSG mice were implanted with patient-derived xenografts (PDXs; four mice per group) from triple-negative wild type (WT) or mutated p53 (MUT) breast tumors and treated intraperitoneally with a combination of LFM (20 mg/kg) and iChk1 (20 mg/kg). **d** Balb/c mice injected with syngeneic 4T1 cells (10^6^ cells per animal; 5–6 mice per group) into mammary fat pad were treated intraperitoneally with LFM (20 mg/kg) alone or in combination with iChk1 (20 mg/kg)—see Methods for details. 4T1 cells circulating in blood or metastatic to lungs and liver were isolated and a number of 4T1 colonies was counted—see Methods for details. **e** Scheme of the mechanism of DHODH-induced cell cycle arrest. In **a**–**d**, data are shown as mean ± SEM. *P < 0.05, two-way ANOVA.

To establish the effect of combined DHODH/Chk1 inhibition on tumor growth in vivo, FVB/N *c-neu* mice were injected with murine breast cancer NeuTL p53-deficient cells and administered with leflunomide and the Chk1 inhibitor intraperitoneally twice a week for 2 weeks (see Methods for details). In parallel, transgenic FVB/N *c-neu* mice with spontaneous Her2^high^, wt p53 breast carcinomas (Supplementary Fig. 5J) were treated using the same regimen. Similarly as for in vitro experiments, we observed reduced growth of p53-deficient tumors treated with the combination of leflunomide and the Chk1 inhibitor compared with the leflunomide treatment alone (Fig. 6a), while spontaneous wt p53 tumors did not show any additional benefit of combined administration (Fig. 6b). To corroborate these findings in a clinically relevant model, we used mice with patient-derived xenografts (PDXs) originating from triple-negative breast cancers (TNBC) with either wt p53 or mutant p53 (Supplementary Fig. 5K). Interestingly, we observed strong inhibition of p53-mutant tumors treated together with leflunomide and Chk1 inhibitor, while only moderate effect was apparent for the wt p53 tumors (Fig. 6c).

Loss of functional p53 often results in promotion of metastases. Therefore, Balb/c mice were transplanted with metastatic breast cancer 4T1 cells into the mammary fat pad and treated with the agents twice a week for 2 weeks. At the end of the treatment, circulating tumor cells in blood and metastases in lungs and liver were evaluated (see Methods for details). In contrast to untreated and Chk1 inhibitor-treated mice, very few metastases were observed in the lungs and the liver in mice treated with leflunomide alone, while none were observed in mice undergoing the combinatorial treatment (Fig. 6d, Supplementary Fig. 6A; see Supplementary Fig. 6B for primary tumor growth). Further, DHODH inhibition resulted in the induction of senescence (Supplementary Figs. 1A, D, 4D and 6C), and the associated increase of pro-inflammatory cytokines, in tumor cells (Supplementary Fig. 6D). Decreased motility associated with senescence^29^ could explain the ability of leflunomide to effectively reduce metastasis, but not solid tumors (Supplementary Fig. 6A, B).

## Discussion

Pyrimidine nucleotides play a critical role in cellular physiology and metabolism, mainly by providing precursors for RNA and DNA synthesis^30^. While pyrimidine nucleotides can be synthesized via the de novo pyrimidine biosynthetic pathway dependent on the respiration-linked DHODH, they can be also formed by salvage pathways in a DHODH-independent manner. The relative contribution of de novo pyrimidine synthesis to maintaining the pyrimidine pool depends on the cell type and the developmental stage. It is less important in resting or fully differentiated cells, where salvage pathways are sufficient^31^. In contrast, de novo pyrimidine synthesis gains prominence in rapidly proliferating cells due to an increased demand for nucleic acid precursors resulting from a high rate of DNA replication and nucleic acid biosynthesis^32^.

We have recently demonstrated the crucial importance of DHODH-dependent de novo pyrimidine synthesis for the ability of cancer cells to proliferate and form tumors^20^. Similarly, administration of leflunomide in mice reduced tumor growth in this report, in agreement with the findings of others^6,33^.

Cell cycle arrest induced by DHODH inhibition/deficiency is likely to prevent proliferation while not inducing cell death, which may compromise its effect on cancer therapy. Therefore, it is warranted to define factors that promote cell death upon DHODH inhibition. Indeed, despite high expectations, targeting DHODH was shown to be therapeutically effective only in some types of cancer. For example, such intervention was successful in patients with TNBC, where inhibition of de novo pyrimidine synthesis sensitized the tumors to genotoxic therapy^8^, similar as reported for patients with acute myeloid leukemia^9,33^ or multiple myeloma^34^. Better understanding of molecular mechanisms sensitizing to DHODH inhibition may therefore help to translate DHODH-directed therapy into more efficient clinical outcomes.

In the present study, we identified the combination of stalled replication and ribosomal stress, induced either by DHODH deficiency/inhibition or respiratory insufficiency, both of which result in the failure to produce pyrimidines^20^, as major factors regulating both cell cycle arrest and cell death in response to DHODH inhibition/deficiency. The lack of balanced dNTP pool can induce delay of replication fork progression and DNA replication as well as inhibition of RNA synthesis and proper assembly of ribosome^35,36^. In accordance with previous reports^5,7,37,38^, we have observed cell cycle arrest in the early S phase after inhibition of DHODH activity by leflunomide, a clinically used agent in patients with rheumatoid arthritis and multiple sclerosis^39,40^. Surprisingly, we did not observe an increase in DNA damage, unlike reported previously^41^. In contrast, we observed accumulation of RPA as well as BrdU on ssDNA, indicating replication stress triggered by defective replication fork recovery as a consequence of pyrimidine deficiency. Replication stress explains the presence of activated checkpoints and induction of p53 in the absence of DNA damage. The mechanism of activation relies on binding of RPA proteins to ssDNA, which is recognized by its binding partner ATR-interacting protein (ATRIP) and is activated by topoisomerase IIβ-binding protein 1 (TopBP1). This complex then recruits and activates ATR, which phosphorylates and activates ATM^42,43^. ATR/ATM activation after replication stress was shown to arrest cells in S/G2^42^). Indeed, leflunomide treatment inhibits transcriptional elongation of genes required for melanoma growth^44^, which corresponds to our observation of stalled replication.

Uncontrolled proliferation of cancer cells requires extensive protein synthesis dependent on constant supply of newly formed ribosomes. Therefore, depletion of the pyrimidine nucleotide pool leads to ribosomal stress since UTP is essential for ribosome biogenesis. Our results confirmed decreased expression of ribosome-related genes and proteins in DHODH-dysfunctional cells. Aberrant expression of ribosomal genes results in nucleolar stress with ensuing cell cycle arrest^45^. This is linked to the nucleolus as a stress sensor, coordinating p53 activation via a number of proteins such as nucleophosmin (B23), RPL5, and RPL11, which directly bind to MDM2 and prevent ubiquitin-mediated p53 degradation^46^. While p53 can be pro-apoptotic, stabilization of p53 after DHODH inhibition represents an important checkpoint ensuring cell cycle arrest and cell survival after pyrimidine depletion. Removal of this checkpoint leads to increased cell death, a consequence of damage induced by attempts to proceed in the cell cycle without complete replication.

Inactivation of the p53 tumor suppressor function is a frequent event in tumorigenesis, giving rise to cell cycle progression and uncontrolled proliferation. Since suppression of p53 led only to a modest increase in cell death and we observed cell cycle arrest also in p53-deficient cells, it is likely that inhibition of de novo pyrimidine synthesis leads to p53-independent cell cycle arrest. Conventional chemotherapeutics have been combined with Chk1 inhibition in order to increase cytotoxicity^47,48^. Chk1, phosphorylated by ATR, prevents replication of damaged DNA and prevents premature chromosome condensation and cell division^49^. Ineffective activation of the ATR/Chk1 pathway, as in PTEN-deficient tumors, therefore increases their sensitivity to depletion of pyrimidine pool^6^. Using a selective Chk1 inhibitor LY2603618, currently undergoing phase II clinical trial^48^, we have observed increased elimination of tumor cells lacking de novo pyrimidine synthesis, especially those with p53-deficient status.

Importantly, we observed a synergistic effect of DHODH and Chk1 inhibition on tumor progression in vivo as well as its effect on metastases. Such treatment was also highly effective in PDX tumor models derived from patients with p53-mutant triple-negative breast cancer, contrary to those derived from wt p53 breast cancer showing its clinical relevance. Decreased motility associated with senescence induced after DHODH inhibition in tumor cells could explain the anti-metastatic effect of leflunomide. However, increased production of pro-inflammatory cytokines by senescent cells increase the risk of chronic inflammation and secondary tumor formation^50^ (Supplementary Fig. 6C), and their persistence in the organism is detrimental. Elimination of tumor cells, not only inhibition of proliferation leading to senescence, is therefore preferred for effective therapy. Combination of DHODH and Chk1 inhibitors is clearly superior to DHODH inhibition alone in this respect and represents a novel approach to treatment of tumors that are poorly responding to established therapies.

In our in vivo experiments, the combination of DHODH/Chk1 inhibition was non-toxic. However, previous studies using teriflunomide, the biologically active metabolite of leflunomide, showed high toxicity in mice indicating that the use of teriflunomide in patients may be unrealistic^41^. It should be noted that leflunomide is gradually converted to teriflunomide ablating the toxic effects of the latter when administered at high doses^51^. Moreover, mitochondrial localization of DHODH allows for targeting of leflunomide to mitochondria using a specific anchor, which could further increase the tumor sensitivity to this drug while minimizing its effects on normal cells.

In conclusion, we present in detail the complex machinery linked to cell cycle inhibition after suppression of pyrimidine synthesis. It comprises activation of two parallel inhibitory pathways, RPL/MDM2-modulated p53 stabilization and Chk1 activation, in response to replication and ribosomal stress induced by the depletion of pyrimidines (Fig. 6e). This explains why p53-deficient cells are more sensitive to Chk1 inhibition, since absence of both pathways allows for tumor cells to re-enter the cell cycle without replicated DNA, which in turn results in aberrant mitosis and cell death. Since p53 is mutated in more than 50% of tumors, simultaneous inactivation of DHODH and inhibition of the Chk1 kinase appears as a promising therapeutic modality for patients with hard-to-manage p53-mutant tumors.

## Methods

### Chemicals and antibodies

Leflunomide (L5025), nocodazole (M1404), uridine (U3003), etoposide (E1383), and nutlin 3 (N6287) were purchased from Sigma (St. Louis, MO, USA). The Chk1 inhibitor LY2603618 (S2626) was purchased from Selleckchem (Munich, Germany).

For immunoblotting, the following antibodies were used: anti-RPS6 (#2217), anti-p53 pS15 (#9282), anti-H3 pS10 (#3642), anti-Chk1 pS317 (#2344), anti-Chk2 pT68 (#2661) (all from Cell Signaling Technology, Danvers, MA, USA), anti-Chk2 (sc-9064), anti-Chk1 (sc-8408), anti-RPA32 (sc-56770), anti-Ub (sc-8017), anti-p53 (sc-126), anti-DHODH (sc-166348), anti-UMPS (sc-398086) (all from Santa Cruz Biotechnology, Dallas, TX, USA), anti-VDAC (ab15895) (Abcam, Cambridge, UK), anti-cyclin E (MA5-14336), anti-p21 (701151), anti-MDM2 (700555), B23 (32-5200) (all from ThermoFisher, Waltham, MA, USA). HPR-conjugated β-actin (PA1-183-HRP), HPR-conjugated α-tubulin (MA5-16308-HRP) (ThermoFisher, Waltham, MA, USA), anti-GAPDH (#5174) (all from Cell Signaling Technology, Danvers, MA, USA) or anti-TFIIH (sc-271500) (Santa Cruz Biotechnology) were used as loading controls. All antibodies were diluted 1:1000 in 2.5% non-fat milk. IgG-HRP anti-rabbit (170-6515) and anti-mouse (170-6516) secondary antibodies were purchased from BioRad Laboratories (Hercules, CA, USA). Secondary antibodies were diluted 1:10,000 in 2.5% non-fat milk. The following antibodies were diluted 1:100 in phosphate-buffered saline (PBS): anti-γH2AX (05-636) (Millipore, Billerica, MA, USA), anti-53BP1 (sc-22760) (Santa Cruz Biotechnology) and anti-RPA32 (ab-2175) (Abcam). Secondary antibodies Alexa 488 (anti-rabbit) and Alexa 568 (anti-mouse) were purchased from ThermoFisher (Waltham, MA, USA) were diluted 1:1000 in PBS.

### Cell culture

Human breast carcinoma cells MCF7 and MDA-MB-231, colon adenocarcinoma cells HCT116 (wt p53 and KO p53 cells) and wt p53 mouse melanoma cells B16 were cultivated in DMEM containing 4.5 g/l glucose (Biochrom, Berlin, Germany). All cells are from ATCC. Breast cancer NeuTL cells derived from tumors of transgenic FVB/N *c-neu* mice and 4T1 mouse breast carcinoma cells (ATCC) were cultivated in the RPMI medium containing 4.5 g/l glucose (Biochrom, Berlin, Germany). Media was supplemented with 10% fetal bovine serum (FBS) (Gibco, Carlsbad, CA, USA), 100 U/ml penicillin and 100 μg/ml streptomycin sulfate (Sigma). The RPMI medium was supplemented with sodium pyruvate (1 mM). Cells were kept at 37 °C under 5% CO_2_. All cells were tested for mycoplasma contamination.

### Animal studies

Transgenic FVB/N *c-neu* mice that develop spontaneous tumors at 6–8 months of age were treated with leflunomide (20 mg/kg dissolved in 4% EtOH in corn oil) alone or in combination with Chk1 inhibitor (LY2603618; 20 mg/kg dissolved in 5% DMSO in corn oil) given intraperitoneally twice a week for 2 weeks. In case of combined treatment, leflunomide was applied 24 h before the Chk1 inhibitor. Control mice were treated with the same volume of the excipient (4% ethanol in corn oil or 5% DMSO in corn oil) as was described above for combined treatment. We randomized mice according to the tumor volume before treatment.

Balb/c mice were injected subcutaneously (s.c.) with 10^6^ 4T1 cells in PBS. FVB/N *c-neu* mice (6 weeks old) were injected s.c. with 10^6^ NeuTL cells in PBS before they developed spontaneous tumors. After 1 week (when tumors reached on average volume of 100 mm^3^), mice were treated with leflunomide and the Chk1 inhibitor as described above. At the end of the experiment lungs, liver and blood from Balb/c mice were removed and processed according to the protocol described by Pulaski et al.^52^ to analyze metastases. We randomized mice according to the tumor volume before treatment.

NOD/SCID gamma (NSG) mice were implanted with patient tumor tissue, grown as first-generation xenografts in the mammary fat pad. In brief, mice were anesthetized, the mammary fat pad was surgically exposed and injected with 50 µl of the Matrigel extracellular matrix (Corning, Wiesbaden, Germany). When Matrigel solidified, tumor pieces (~2 mm^3^) were implanted into a pocket excised in the mammary fat pad and secured with an internal stitch. The incision was closed by suture and mice were left on a heated pad until awaken. When tumors reached the volume of ~50 mm^3^, mice (*n* = 3 per group for control mice treated, *n* = 4 per group for treated mice) were treated with the combination of leflunomide (20 mg/kg dissolved in 4% EtOH in corn oil) and the Chk1 inhibitor LY2603618 (20 mg/kg dissolved in 5% DMSO in corn oil) or the excipient (4% ethanol in corn oil and 5% DMSO in corn oil, 100 µl per dose) given intraperitoneally twice a week for 2 weeks. Leflunomide was applied 24 h before the Chk1 inhibitor. We randomized mice according the tumor volume before treatment.

All experiments were approved by the Czech Academy of Sciences Ethics Committee and performed according to the Czech Council guidelines for the Care and Use of Animals in Research and Teaching.

### Evaluation of reactive oxygen species

To assess ROS production, cells were incubated with 2′7′-dichlorofluorescein (DCF; 10 μM; Sigma) for 30 min prior to analysis by flow cytometry using the LSR Fortessa instrument (BD, San Jose, CA, USA). Cells without added DCF probes were used as a control of non-specific signal. Hoechst 33258 (5 μg/ml; Invitrogen, Carlsbad, CA, USA) was added to cells prior to the measurements to exclude dead cells from the analysis.

### Assessment of cell death

The medium containing dead cells was collected into a clear tube, adherent cells were trypsinized, resuspended in the medium containing dead cells and centrifuged at 1000 × *g* for 3 min. The pellet was resuspeneded in 200 μl of annexin V-binding buffer containing 0.3 µl of annexin V-Dyomics 647 (Apronex, Vestec, Czech Republic), and incubated for 20 min at 4 °C. Hoechst 33258 (5 μg/ml) was added before the cells were then analyzed using the LSR Fortessa flow cytometer. Cell death was expressed as the percent of the annexin V-positive/Hoechst-positive fraction.

### SDS-PAGE and immunoblotting

Cells were washed twice with PBS, harvested into Laemmli SDS sample lysis buffer (2% SDS, 50 mM Tris-Cl, 10% glycerol in double distilled H_2_O) and sonicated (2 × 10 s at 1 μm amplitude with 10 s cooling interval) using the Soniprep 150 instrument (MSE, London, UK). Protein concentration was estimated using the BCA method (Pierce Biotechnology, IL, Rockford, USA). Cell lysates were supplemented with 100 mM DTT (Sigma) and 0.01% bromophenol blue (Sigma) before separation by SDS-PAGE. The same amount of protein (50–70 μg) was loaded into each well. The protein was transferred onto a nitrocellulose membrane using wet transfer and detected by specific antibodies combined with horseradish peroxidase-conjugated secondary antibodies (goat anti-rabbit or goat anti-mouse). Peroxidase activity was detected using the ECL Western Blotting Substrate or the SuperSignal West Femto Extended Duration Substrate (Thermo Fisher).

For RPA protein detection, cells were incubated for 10 min in the pre-extraction buffer (0.5% Triton X-100, 20 mM HEPES pH 7.4, 50 mM NaCl, 3 mM MgCl_2_, 1 mM PMSF) before harvesting into Laemmli SDS sample lysis buffer.

### Immunoprecipitation

Cells grown to near confluency in 10 cm^2^ plates were washed twice with cold PBS, harvested into 300 μl of the harvest buffer (100 mM HEPES pH 7.5, 500 mM NaCl, 0.5% Nonidet P40, 2% glycerol, 2 mM EDTA, protease and phosphatase inhibitor cocktail) and incubated for 30 min on ice. The suspension was centrifuged for 15 min/4 °C/13,200 r.p.m., the lysate was transferred into a clean tube and protein concentration was assessed using the BCA kit (Pierce Biotechnology). Protein A/G-sepharose beads (10 μl) were gently washed in 500 μl of the harvest buffer and centrifuged (1 min/room temperature (RT)/3000 r.p.m.). Clear beads were mixed with 500 μg of the lysate and incubated using a vertical shaker for 1 h at 4 °C to remove unspecific binding. After pre-incubation, beads were removed from the sample (1 min/RT/3000 r.p.m.), and the samples were incubated with the primary antibody overnight at 4 °C. Thirty microlitres of clear beads were then incubated with the sample for 3 h using a vertical shaker. After incubation, beads were centrifuged (1 min/RT/3000 r.p.m.), the supernatant was removed, beads were mixed with 2× Laemli Buffer (see SDS-PAGE) and 100 mM DTT + bromphenol blue, and denatured at 95 °C for 5 min. The samples were centrifuged, the supernatant transferred into a clean tube and the samples were then processed as described in the section on SDS-PAGE and immunoblotting.

### Detection of senescence-associated β-galactosidase activity

SA-β-gal activity was detected as previously described^53^ with slight modifications. Cells were washed with PBS, fixed with 0.5% glutaraldehyde (in PBS; pH 7.2; Sigma) and washed with PBS (pH 6.0) supplemented with 1 mM MgCl_2_. Cells were stained with the X-gal solution (1 mg/ml X-gal, 0.12 mM K_3_Fe[CN]_6_, 0.12 mM K_4_Fe[CN]_6_, 1 mM MgCl_2_ in PBS at pH 6.0) at 37 °C for 3–5 h. The signal was detected using the Leica DMIL Led microscope.

### Indirect immunofluorescence

Cells grown on glass coverslips were fixed with 4% formaldehyde and permeabilized with 0.1% Triton X-100 in two consecutive steps, each at room temperature for 15 min. After washing with PBS, cells were incubated in 10% FBS (diluted in PBS) for 30 min to block unspecific signals. After this step, cells were incubated with diluted primary antibodies (1 h, RT), washed extensively with PBS/0.1% Tween 20 and incubated with secondary antibodies (1 h, RT). To counterstain nuclei, coverslips were mounted in Mowiol containing 4′,6-diamidino-2-phenylindole (DAPI; Sigma) and viewed in a confocal microscope (Leica SP8). For detection of the bound RPA protein, cells were washed for 15 min in the pre-extraction buffer (0.5% Triton X-100, 20 mM HEPES pH 7.4, 50 mM NaCl, 3 mM MgCl_2_, 1 mM PMSF, 0.01 M β-glycerol phosphate) before fixation in formaldehyde to wash out all unbound RPA.

### siRNA-mediated gene knock-down

Cells were transfected with siRNAs using Lipofectamine RNAiMAX (Invitrogen) following the manufacturer´s instructions. siRNAs against RPL5 (sense sequence: 5′-GAC GAG AGG GUA AAA CUG Att-3′), RPL11 (sense sequence: 5′-GGU GCG GGA GUA UGA GUU Att-3′), RPS6 (sense sequence: 5′-CCU UAA AUA AAG AAG GUA Att-3′) and p53 (sense sequence: 5′-GGU GAA CCU UAG UAC CUA Att-3′), DHODH#1 (sense sequence: 5′-GGU AUG GAU UUA ACA GUC Att-3′), DHODH#2 (sense sequence: 5′-CGG GAU UUA UCA ACU CAA Att-3′), UMPS#1 (sense sequence: 5′- GCA GAU GCU UUA GGA CCU Att-3′), and UMPS#2 (sense sequence: 5′- GUA UGA AGG AGG UAU CUU Utt-3′) were purchased from ThermoFisher (Waltham, MA, USA). Non-targeting siRNA (Silencer® Select Negative Control No. 1, #4390843) was used as a negative control (siNC).

### Quantitative real-time PCR (qRT-PCR)

Total RNA was isolated using RNAzol (400 μl for a 4 cm^2^ dish; Molecular Research Center, Cincinnati, OH, USA). First-strand cDNA was synthesized from 1 μg of total RNA with random hexamer primers using Revert Aid First strand cDNA Synthesis Kit (Thermo Fisher). qRT-PCR was performed using the Eco Real-Time PCR System (Illumina, San Diego, CA, USA) with 5× HOT FIREPol Evagreen qPCR Supermix GreenE dye (Solis Biodyne, Tartu, Estonia). The relative quantity of cDNA was estimated by the ΔΔCT method; data were normalized to β-actin. The following primers were purchased from Sigma: RPL5: 5′-CCA AAT ACA GGA TGA TAG TTC GTG-3′, 5′-TTG GCA GTT CGT GTG CAT ACG C-3′; RPL11: 5′-AGA GTG GAG ACA GAC TGA CGC G-3′, 5′-CGG ATG CCA AAG GAT CTG ACA G-3′; 45S: 5′-CTC CGT TAT GGT AGC GCT GC-3′, 5′-GCG GAA CCC TCG CTT CTC-3′; 18S: 5′-ACC CGT TGA ACC CCA TTC GTG A-3′, 5′-GCC TCA CTA AAC CAT CCA ATC GG-3′; mouse IL6: 5′-TAC CAC TTC ACA AGT CGG AGG C-3′, 5′-CTG CAA GTG CAT CAT CGT TGT TC-3′; mouse IL8: 5′-CTC TAT TCT GCC AGA TGC TGT CC-3′, 5′-ACA AGG CTC AGC AGA GTC ACC A-3′; mouse TNFa: 5′-GCC TCT TCT CAT TCC TGC TTG-3′, 5′-CTG ATG AGA GGG AGG CCA TT-3′; mouse β-actin: 5′- CAT TGC TGA CAG GAT GCA GAA GG-3′, 5′-TGC TGG AAG GTG GAC AGT GAG G-3′; β-actin: 5′-CCA ACC GCG AGA AGA TGA-3′, 5′-CCA GAG GCG TAC AGG GAT AG-3′. Data are expressed as mean values ± SEM of a minimum of three independent experiments performed in triplicates. The *P* values were calculated using two-way ANOVA; differences with *P* < 0.05 were considered statistically significant.

### Knock out and reconstitution of DHODH in 4T1 cells

To prepare DHODH^KO^ cells, we used the CRISPR/Cas9 system^54^. Guide RNAs sequences from the GeCKO library (v2 09Mar2015) were synthesized and cloned into *Bsm*BI-cleaved pXPR_001 vector containing the mammalian codon-optimized Cas9 nuclease expression cassette. Parental 4T1 cells were transiently transfected using Lipofectamine 3000 (Thermo Fisher), grown for 24 h and selected with puromycin for 48–72 h, or until all non-transfected control cells were eliminated. Surviving cells were sorted into 96-well plates, expanded, and clones unable to grow in media lacking uridine and pyruvate (for DHODH^KO^ cells) were selected. The absence of DHODH was verified by western blotting. The guide RNA sequence used was as follows: 5′-TCA GGT ACT CGG CGT AGA AA-3′.

To reconstitute DHODH protein expression, the DHODH coding sequence was PCR-amplified from cDNA obtained from mouse B16 cells. Primers used were 5′-ATA AAG AAT TCC ACC ATG GCG TGG AGA CAG CTG-3′ (forward) and 5′-ATA AAG GAT CCT CAC CTG CGG TGA TCT ACT C-3′ (reverse). The gel-purified PCR product was cleaved by fast-digest *Eco*RI and *Bam*HI enzymes (Thermo Fisher) and ligated into the pCDH-CMV-MCS-EF1-Puro lentiviral vector (System Biosciences) digested in the same way. Lentiviral particles were produced as described^55^ and used to transduce 4T1 DHODH^KO^ cells, followed by puromycin selection. Expression of the DHODH protein was verified by western blotting.

### DHODH activity assay

DHODH activity was evaluated using a modified protocol according to Yin et al.^56^. In brief, cells were collected, washed with PBS, re-suspended in potassium phosphate buffer (0.1 M, pH 7.0) and lysed using three freeze-thaw cycles; the lysates were incubated in the solution of 160 mM K_2_CO_3_/HCl (pH 8.0), 400 μM dihydroorotate, 80 μM decylubiquinone at 37 °C for 60 min. The reference sample was kept on ice. The reaction mixture was supplemented with 20 mM K_2_CO_3_, 2 mM K_3_[Fe(CN)_6_] and 1 mM 4- (trifluoromethoxy)benzamidoxime (4-TFMBAO), and heated at 80 °C for 4 min. The reaction was stopped by cooling on ice, and fluorescence intensity was measured using the Tecan Infinite M200 plate reader (Schoeller; Prague, Czech Republic); the excitation and emission wavelengths were 320 and 420 nm, respectively. The calibration curve was plotted using lysate-free samples containing 0.5, 0.75 and 1 μM orotic acid.

### Cell cycle analysis

For cell cycle analysis, cells were pre-treated with 10 μM nocodazole for 16 h at 37 °C to allow their accumulation in G2/M. Cells were trypsinized, harvested into 500 μl of PBS and centrifuged (2500 r.p.m./3 min). Pellets were resuspended in 70 μl of PBS, fixed with 500 μl of cold 80% EtOH added drop-wise during shaking and incubated at −20 °C for 30 min. EtOH was then removed, cells were re-suspended in 200 μl of PSB containing 2.5 μg/ml propidium iodide (for 10^6^ cells) and the samples were analyzed by flow cytometry. For BrdU detection, 2 M HCl was added for 30 min (room temperature) after fixation to denaturate DNA. HCl was removed and the pellet neutralized with 100 μl of 0.1 M disodium tetraborate (pH 8.5) for 1 min. Cells were then incubated for 1 h with anti-BrdU-FITC IgG (BD Biosciences; 0.5 μg/ml of PBS) in the dark. Finally, cells were re-suspended in 200 μl of PSB containing 2.5 μg/ml propidium iodide and analyzed by flow cytometry.

### Microarray analysis

Transcriptomic analysis of parental, D0, D5, D10, D15, D20 and D25 4T1 cells was performed using a standard protocol and the MouseGene 2.0 ST array (Affymetrix; Thermo Fisher). The data are deposited under the accession number MTAB-6150 in the EBI ArrayExpress database.

### Statistical analysis

Unless stated otherwise, data are mean values ± SEM of at least three independent experiments. In mouse experiments, groups of six animals were used, unless stated otherwise. Two-way Annova presented as mean ± standard error of means (SEM) was used to assess statistical significance with *P* < 0.05 being regarded as significant, using GraphPad Prism software. Images are representative of at least three independent experiments.

## Supplementary information

Supplementary Table 1

Supplementary Table 2

Supplementary Table 3

Supplementary Fig. 1

Supplementary Fig. 2

Supplementary Fig. 3

Supplementary Fig. 4

Supplementary Fig. 5

Supplementary Fig. 6

Supplementary Figure Legends
