# Supplementary figures and images for: Replication and ribosomal stress induced by targeting pyrimidine synthesis and cellular checkpoints suppress p53-deficient tumors
Source: Cell Death Dis. 2020 Feb 7;11(2):110. doi: 10.1038/s41419-020-2224-7 (PMC7007433; doi:10.1038/s41419-020-2224-7)

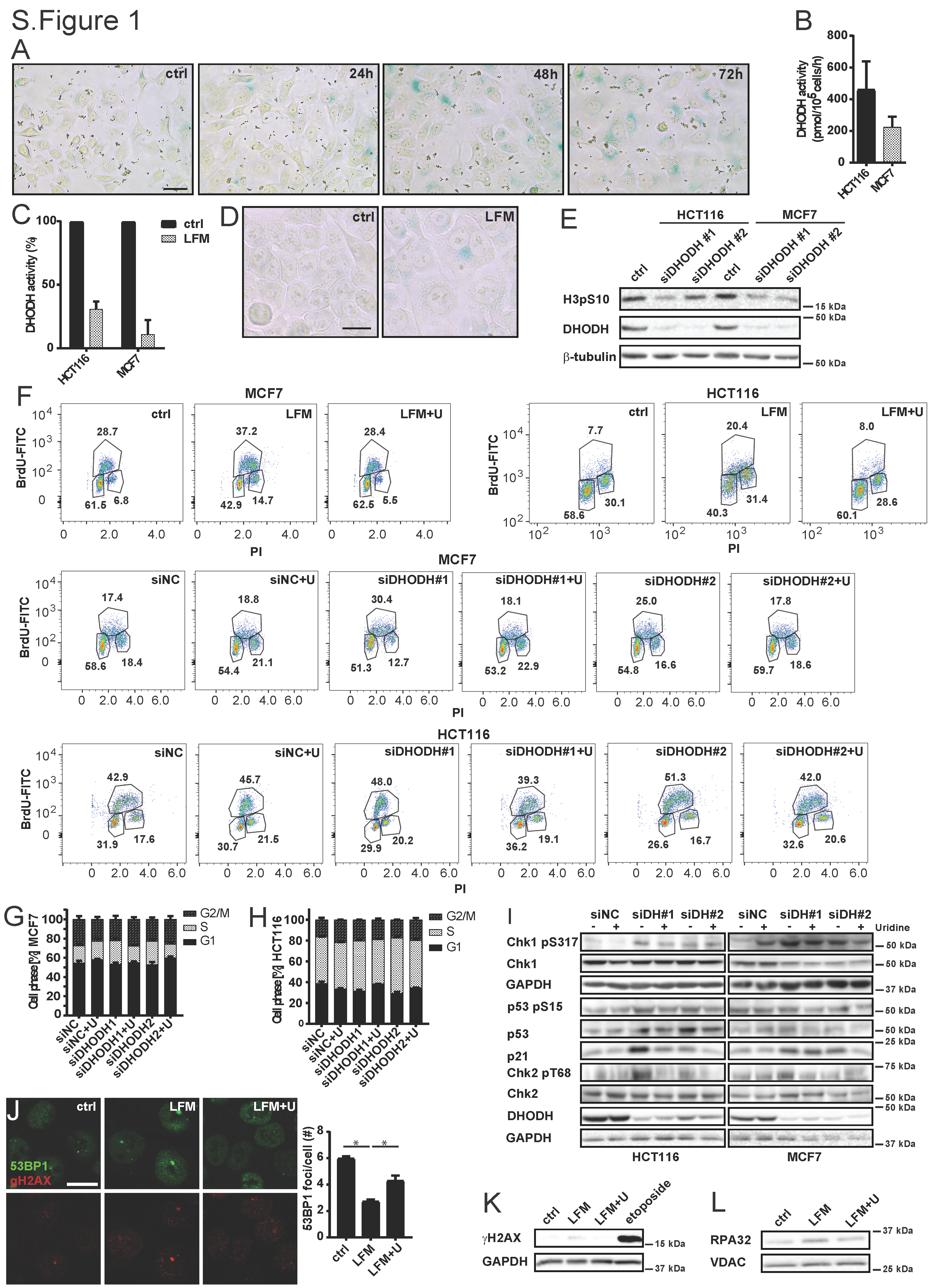

Supplement: Supplementary file 4 — Supplementary Fig. 1 [file 41419_2020_2224_MOESM4_ESM.png]

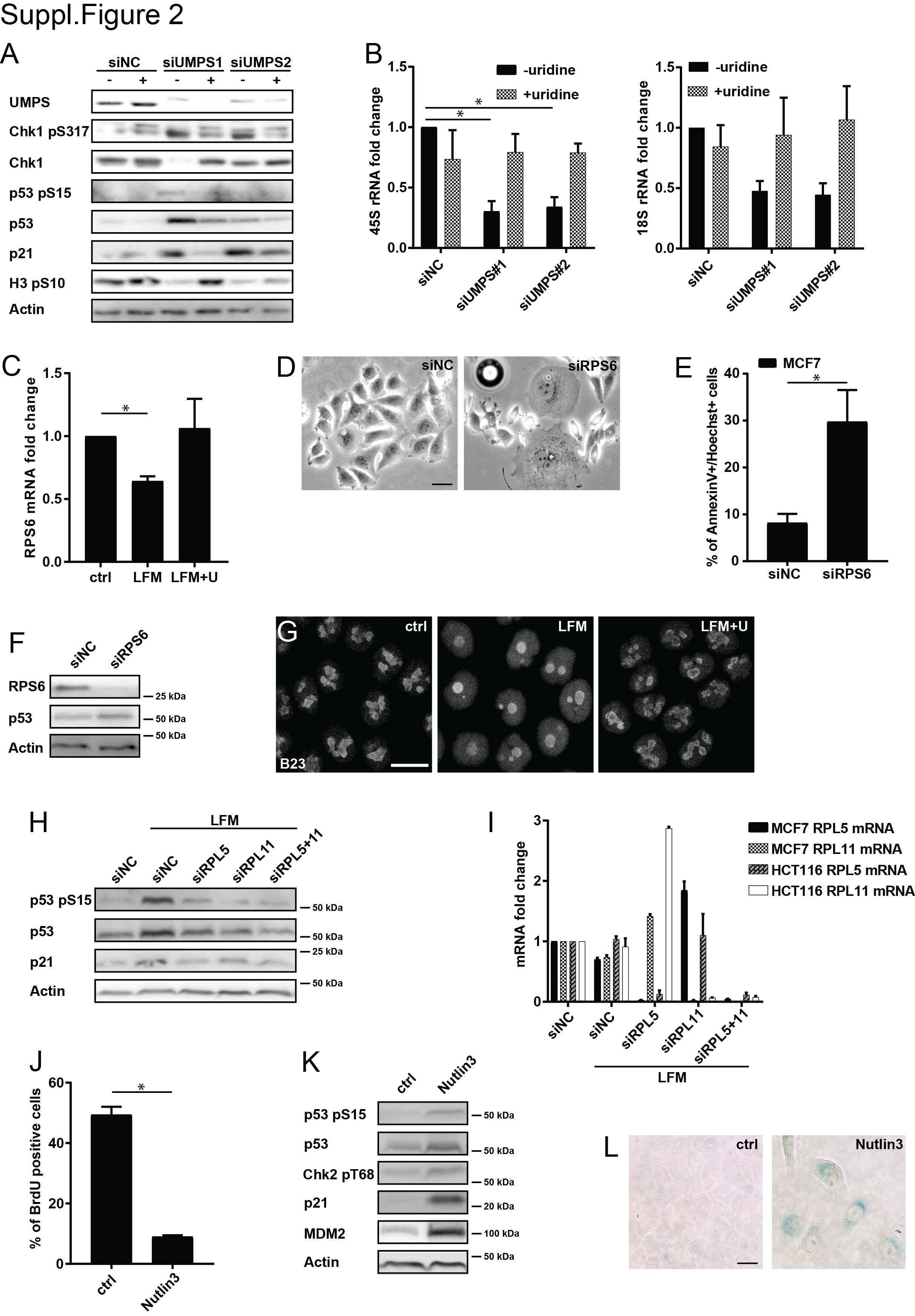

Supplement: Supplementary file 5 — Supplementary Fig. 2 [file 41419_2020_2224_MOESM5_ESM.png]

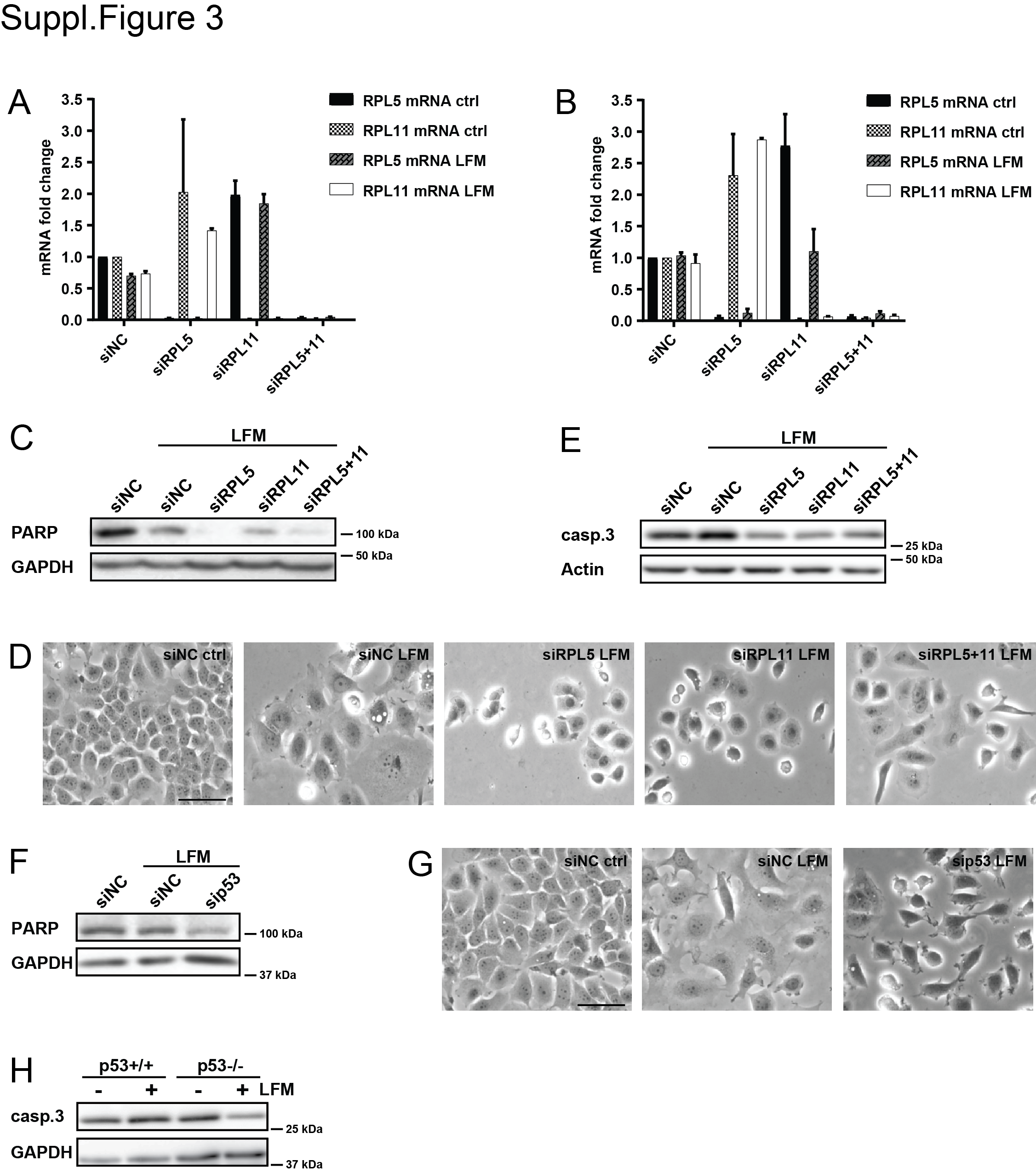

Supplement: Supplementary file 6 — Supplementary Fig. 3 [file 41419_2020_2224_MOESM6_ESM.png]

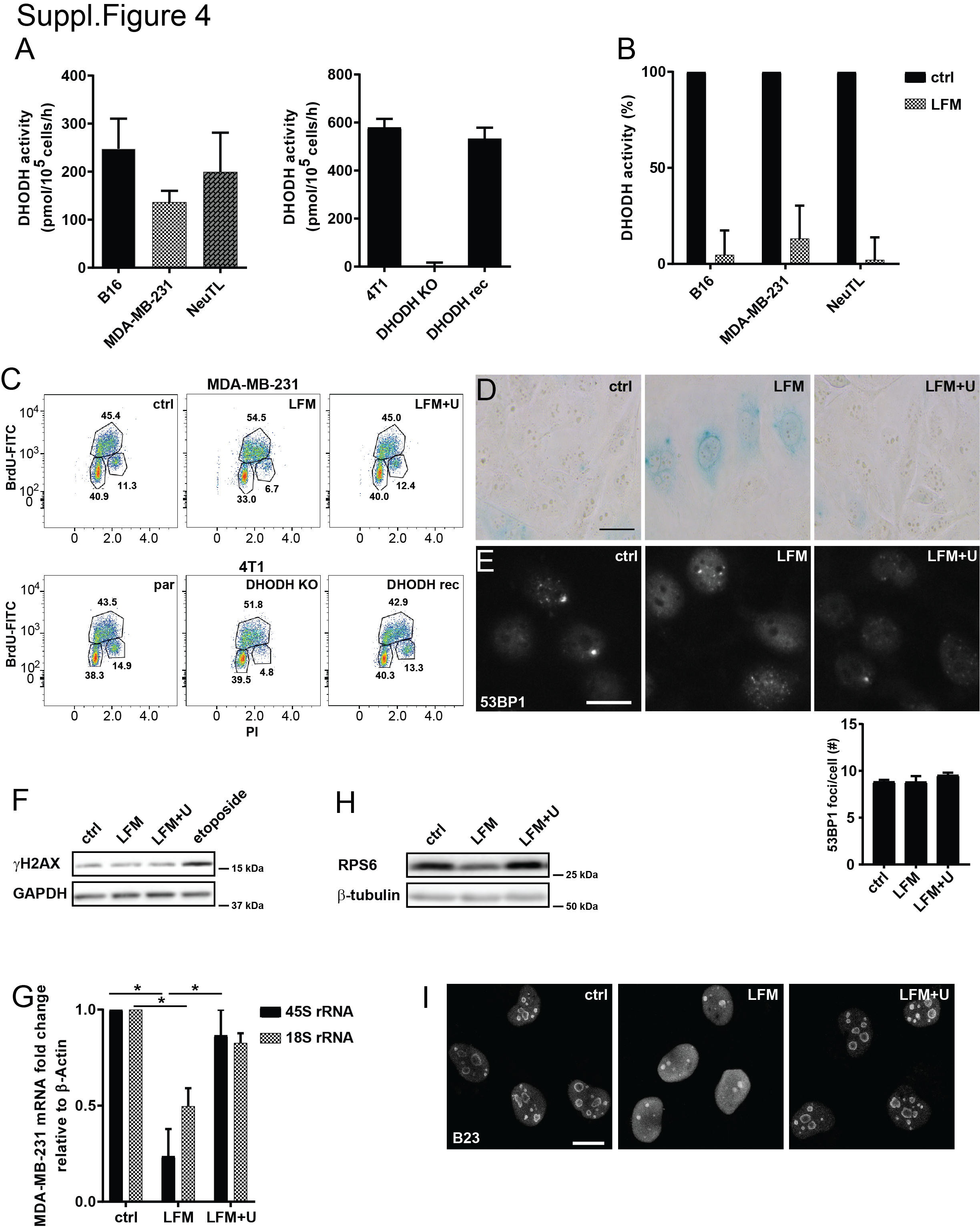

Supplement: Supplementary file 7 — Supplementary Fig. 4 [file 41419_2020_2224_MOESM7_ESM.png]

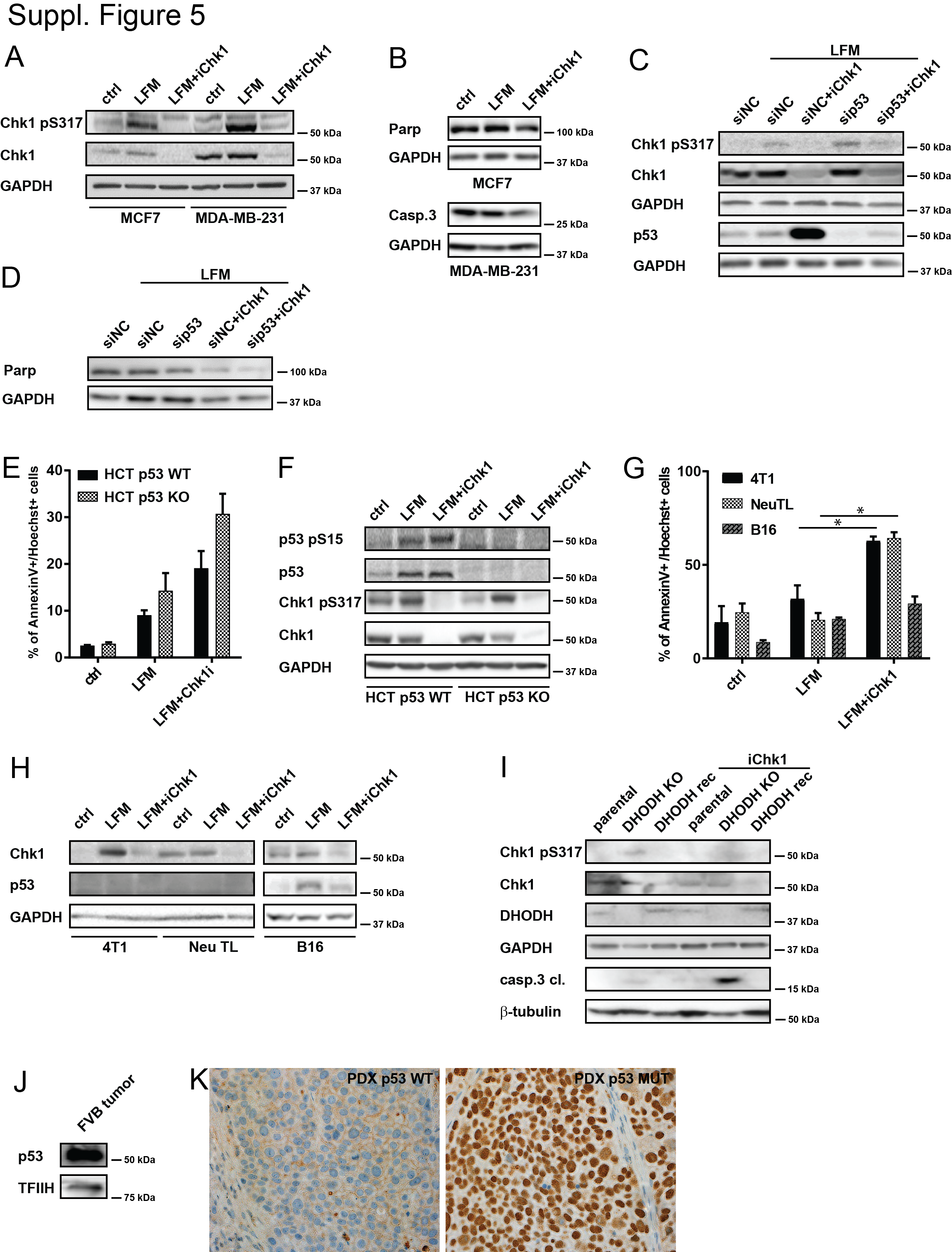

Supplement: Supplementary file 8 — Supplementary Fig. 5 [file 41419_2020_2224_MOESM8_ESM.png]

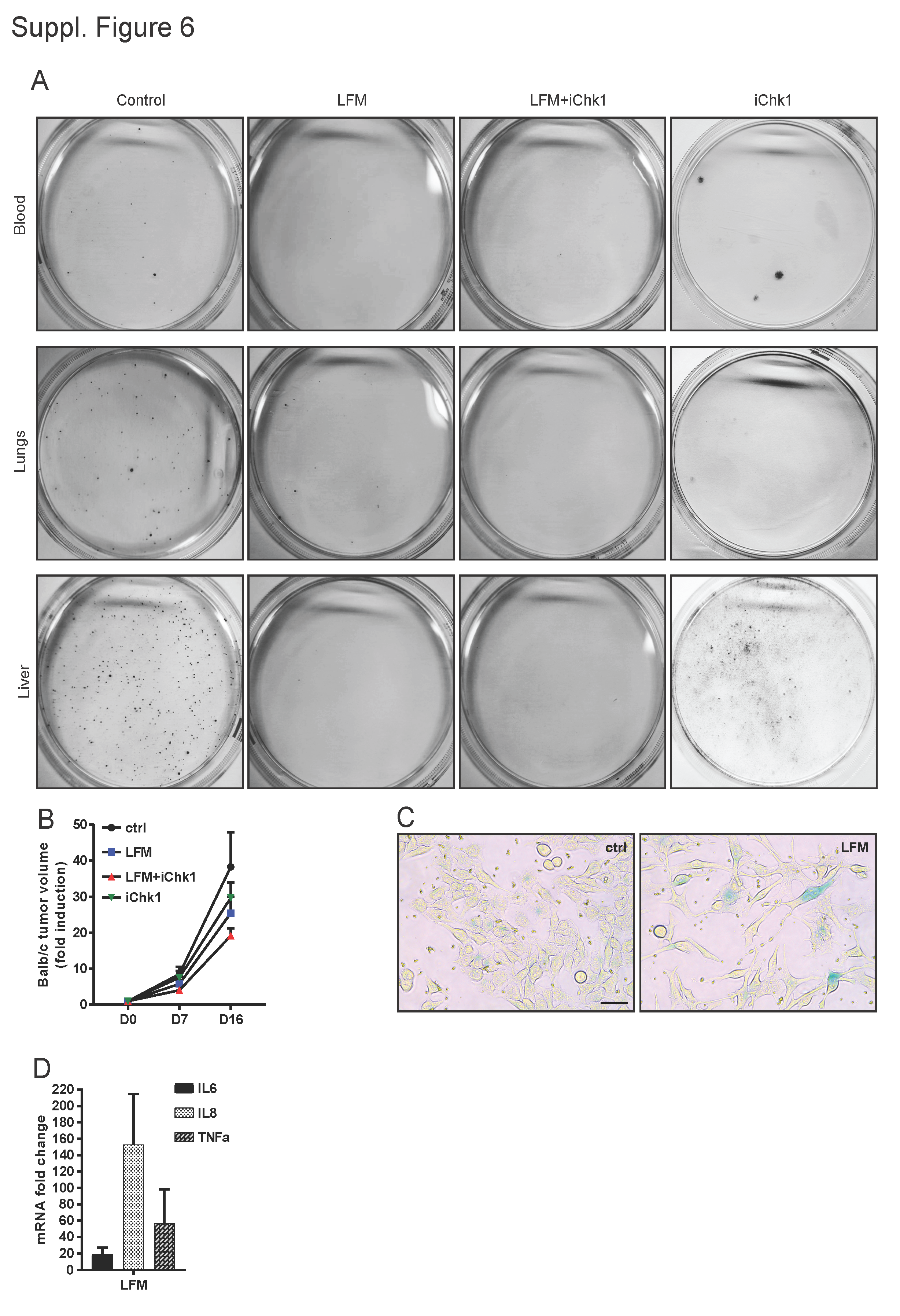

Supplement: Supplementary file 9 — Supplementary Fig. 6 [file 41419_2020_2224_MOESM9_ESM.png]
