# Supplementary material for: Replication and ribosomal stress induced by targeting pyrimidine synthesis and cellular checkpoints suppress p53-deficient tumors
Source: Cell Death Dis. 2020 Feb 7;11(2):110. doi: 10.1038/s41419-020-2224-7 (PMC7007433; doi:10.1038/s41419-020-2224-7)
Supplement: Supplementary file 10 — Supplementary Figure Legends [file 41419_2020_2224_MOESM10_ESM.docx]

**Supplementary Figure Legends**

**Supplementary Figure 1/1:** DHODH induced replication stress

(A) Detection of β-galactosidase activity in MCF7 cells exposed to LFM (50 µM) as indicated. Scale bar 50 µm. (B) Assessment of DHODH activity in HCT116 and MCF7 cells. (C) HCT116 and MCF7 cells were exposed to LFM (50 µM) overnight and changes in the DHODH activity in control and treated cells were evaluated. (D) Detection of β-galactosidase activity in HCT116 cells exposed to LFM (50 µM) for 72 h. Scale bar 50 µm. (E) Immunoblot detection of DHODH and H3pS10 in HCT116 and MCF7 cells with silenced DHODH (siDHODH) using specific siRNA. Non-targeting siRNA (siNC) was used as a control. β-tubulin was used as a loading control. (F) Representative flow cytometry dot plots in MCF7 and HCT116 cells exposed to leflunomide (LFM; 50 µM) for 72 h, or with silenced DHODH in the presence or absence of uridine (U; 50 µg/l). (G) Assessment of cell cycle distribution in MCF7 and (H) HCT116 cells with silenced DHODH (siDHODH) using specific siRNA and in the presence or absence of uridine (U; 50 µg/l). Non-targeting siRNA (siNC) was used as a control. The asterisk indicates significanct differences in S-phase changes. In B, C G, H, data are shown as mean ± SEM n=3. *P<0.05, two-way ANOVA. In other panels, representative experiment (form total number of 3 experiments) is shown.

**Supplementary Figure 1/2:** DHODH induced replication stress

(I) Immunoblot detection of total and serin 317-phosphorylated Chk1, total and serin 15-phosphorylated p53, total and threonine 68-phosphorylated Chk2, p21 and DHODH in HCT116 and MCF7 cells with silenced DHODH using specific siRNA in the presence or absence of uridine (U; 50 µg/l). Non-targeting siRNA (siNC) was used as a control. GAPDH was used as a loading control. (J-L) HCT116 cells were exposed to LFM (50 µM) for 72 h in the presence or absence of uridine (U; 50 µg/l). (J) Immunofluorescence detection of 53BP1 and γH2AX. Scale bar 15 µm. (L) Immunoblotting detection of γH2AX protein level relative to β-tubulin. (L) Immunoblotting detection of RPA32 protein level relative to VDAC. In J, data are shown as mean +/- SEM, n=3; *P<0.05, two-way ANOVA. In other panels, representative experiment (form total number of 3 experiments) is shown.

**Supplementary Figure 2:** DHODH inhibition induce ribosomal stress

(A-B) HCT116 cells were treated in the presence or absence of uridine (U; 50 µg/l) with specific siRNA to downregulate UMPS (siUMPS). Non-targeting siRNA (siNC) was used as a control. (A) Immunoblot detection of UMPS, Chk1 pS317, Chk1, p53 pS15, p53, p21 and H3 pS10. β-actin was used as a loading control. (B) Expression of 18S and 45S rRNA was measured by qRT-PCR. (C) MCF7 cells were exposed to leflunomide (50 µM) for 72 h in the presence or absence of uridine (U; 50 µg/l). Expression of RPS6 was assessed by qRT-PCR. (D-F) MCF7 cells were tranfected with specific siRNA against RPS6 or with non-targeting siRNA (siNC), respectively. (D) Cell morphology was displayed using phase contrast microscopy. Scale bar 20 µm. (E) Cell death was evaluated by annexin V/Hoechst positivity using FACS. (F) Immunoblot detection of RPS6 and p53. β-actin was used as a loading control. (G) Immunofluorescent detection of B23 in HCT116 cells exposed to LFM (50 µM) for 72 h in the presence or absence of uridine (U; 50 µg/l). Scale bar represents 15µm. (H-I) MCF7 and HCT116 cells were exposed to LFM (50 µM) for 72 h after downregulation of RPL5, RPL11 alone or in combination using specific siRNA. (H) Protein level of p53 pS15, p53 and p21 was detected in MCF7 cells by immunoblot. β-actin was used as a loading control. (I) mRNA levels of RPL5 and RPL11 were detected using qRT-PCR. (J-L) MCF7 cells were treated with Nutlin3 (5µM) for 24 h and (J) quantitative analysis of BrdU incorporation was assessed. (K) Protein levels of p53 pS15, p53, Chk2 pT68, p21 and MDM2 were detected by immunoblot. β-actin was used as a loading control. (L) β-galactosidase activity was detected. Scale bar 50 µm. In C, D, F and K, data are shown as mean +/- SEM, n=3; *P<0.05, two-way ANOVA. In other panels, representative experiment (form total number of 3 experiments) is shown.

**Supplementary Figure 3:** Inhibition of RPL5 and RPL11 induce cell death in cells with *de novo* pyrimidine depletion

(A, C, D) MCF7 and (B, E) HCT116 cells were exposed to LFM (50 µM) for 72 h after downregulation of RPL5 and RPL11 alone or in combination using specific siRNA. Non-targeting siRNA (siNC) was used as a control. (A, B) RPL5 and RPL11 mRNA levels were quantified by real-time qRT-PCR and (C) PARP and (E) caspase-3 protein levels were detected by western blotting. β-actin or GAPDH were used as a loading control. (D) Morphology of MCF7 cells treated as indicated was displayed using phase contrast microscopy. Scale bar 50 µm. (F) Immunoblot detection of PARP in MCF7 cells exposed to LFM (50 µM) for 72 h after downregulation of p53 using specific siRNA. Non-targeting siRNA (siNC) was used as a control. GAPDH was used as a loading control. (G) Morphology of MCF7 cells exposed to LFM (50 µM) for 72 h after downregulation of p53 using specific siRNA. Non-targeting siRNA (siNC) was used as a control. Scale bar 50 µm. (H) HCT116 wt p53 (p53^+/+^) and HCT116 p53^KO^ (p53^-/-^) cells were exposed to LFM (50 µM) for 72 h and caspase-3 level was detected by immunostaining. GAPDH was used as a loading control. In A and B, data are shown as mean +/- SEM, n=3. In other panels, representative experiment (form total number of 3 experiments) is shown.

**Supplementary Figure 4:** DHODH inhibition induce replication and ribosomal stress in p53 deficient cells

(A) Evaluation of DHODH activity in B16, MDA-MB-231, NeuTL, 4T1, 4T1 DHODH KO and 4T1 DHODH-reconstituted (DHODH rec) cells. (B) B16, MDA-MB-231, NeuTL and 4T1 cells were exposed to LFM (50 µM) overnight and changes in the DHODH activity in control and treated cells were evaluated. (C) Representative flow cytometry dot plots show cell cycle distribution in MDA-MB-231 cells exposed to LFM (50 µM) for 72 h in the presence or absence of uridine (U; 50 µg/l), and in 4T1 DHODH KO and 4T1 DHODH-reconstituted (DHODH rec) cells. (D-I) MDA-MB-231 cells were treated for 72 h with LFM (50 µM) in the presence or absence of uridine (U; 50 µg/l) and (D) senescence-associated β-galactosidase staining was performed. Scale bar 40 µm. (E) Immunofluorescence detection of 53BP1. Scale bar 15 µm. (F) Immunoblotting detection of γH2AX protein level relative to GAPDH. (G) Expression of 45S rRNA and 18S rRNA was measured by qRT-PCR. (H) Level of RPS6 protein in MDA-MB-231 cells treated as indicated was detected by immunoblot. β-tubulin was used as a loading control. (I) Immunofluorescence detection of B23 in MDA-MB-231 cells exposed to LFM (50 µM) for 72 h in the presence or absence of uridine (U; 50 µg/l). Scale bar represents 10 µm. In A, B and G, data are shown as mean +/- SEM, n=3. *P<0.05, two-way ANOVA. In other panels, representative experiment (form total number of 3 experiments) is shown.

**Supplementary Figure 5:** Simultaneous Chk1 and DHODH inhibition sensitizes p53-deficient tumors to cell death

(A, B) MCF7 and MDA-MB-231 cells or (C, D) MFC7 cells with downregulated p53 using specific siRNA were pre-treated with Chk1 inhibitor (iChk1, 5 µM, 30 min) followed by LFM treatment (50 µM, 48 h) and protein levels of Chk1 pS317, Chk1 total and PARP, caspase 3 or p53 was detected by immunoblot. GAPDH was used as a loading control. (E, F) HCT116 wt p53 and HCT116 p53^KO^ cells were pre-treated with iChk1 (5 µM, 30 min) followed by LFM treatment (50 µM, 48 h). (E) Cell death was detected by annexin V/Hoechst positivity using FACS. (F) Protein levels of p53 pS15, p53, Chk1 pS317 and Chk1 was detected by immunoblot. GAPDH was used as a loading control. (G, H) 4T1, NeuTL and B16 cells were pre-treated with iChk1 (5 µM, 30 min) followed by LFM treatment (50 µM, 48 h). (G) Cell death was evaluated by annexin-V/Hoechst positivity using FACS, and (H) protein levels of Chk1 and p53 were detected by immunoblotting. GAPDH was used as a loading control. (I) 4T1 parental, DHODH KO and DHODH reconstituted (DHODH rec) cells were treated with iChk1 (5 µM, 48 h) and immunoblotting detection of Chk1 pS317, Chk1, DHODH and cleaved caspase 3 (casp.3 cl.) was assessed. β-tubulin and GAPDH were used as a loading control. (J) Spontaneous tumors isolated from FVB/N c‑neu mice were homogenized and presence of p53 was detected by immunoblotting. TFIIH was used as a loading control. (K) Immunohistology detection of p53 protein in triple negative breast wt and mutated p53 (MUT) tumors derived from patients. In Eand G, data are shown as mean +/- SEM, n=3; *P<0.05, two-way ANOVA. In other panels, representative experiment (form total number of 3 experiments) is shown.

**Supplementary Figure 6:** Simultaneous Chk1 and DHODH inhibition decrease metastatic potential of p53 deficient cells

(A) Balb/c mice were injected in the mammary gland with syngeneic 4T1 cells (10^6^ cells per animal; 5-6 mice per group) and treated intraperitoneally with LFM (20 mg/kg), iChk1 (20mg/kg) or its combination twice a week for a period of 2 weeks. Crystal violet was used to visualize metastatic 4T1 colonies isolated from the blood, lungs and liver. (B) Balb/c mice injected subcutaneously with syngeneic 4T1 cells (1x10^6^ cells per animal; 5-6 mice per group) were treated intraperitoneally with LFM (20 mg/kg) alone or in combination with iChk1 (20 mg/kg) or with iChk1 (20 mg/kg) alone – see Methods for detail. Tumor volume was evaluated. (C) Detection of β-galactosidase activity in 4T1 cells exposed to LFM (50 µM) for 48 h. Scale bar represents 50 µm. (D) Expression of IL6, IL8 and TNFα in 4T1 cells exposed to LFM (50 µM) for 72h was assessed by qRT-PCR. In D, data are shown as mean +/- SEM, n=3, two-way ANOVA. In other panels, representative experiment (form total number of 3 experiments) is shown.

**Supplementary Table 1: Transcriptome array for cell cycle related genes**

**Supplementary Table 2: Transcriptome array for p53 pathway related genes**

**Supplementary Table 3: Transcriptome array for ribosome related genes**
